# Multiple links between 5-methylcytosine content of mRNA and translation

**DOI:** 10.1186/s12915-020-00769-5

**Published:** 2020-04-15

**Authors:** Ulrike Schumann, He-Na Zhang, Tennille Sibbritt, Anyu Pan, Attila Horvath, Simon Gross, Susan J. Clark, Li Yang, Thomas Preiss

**Affiliations:** 1 EMBL–Australia Collaborating Group, Department of Genome Sciences, John Curtin School of Medical Research, Australian National University, Canberra, 2601 Australian Captial Territory Australia; 2 CAS Key Laboratory of Computational Biology, CAS-MPG Partner Institute for Computational Biology, Shanghai Institute of Nutrition and Health, Shanghai Institutes for Biological Sciences, University of Chinese Academy of Sciences, Chinese Academy of Sciences, Shanghai, 200031 China; 3 Genomics and Epigenetics Division, Garvan Institute of Medical Research, Sydney, 2010 New South Wales Australia; 4 Faculty of Medicine, St Vincent’s Clinical School, University of New South Wales, Sydney, New South Wales 2010 Australia; 5 School of Life Science and Technology, ShanghaiTech University, Shanghai, 201210 China; 6 Victor Chang Cardiac Research Institute, Sydney, New South Wales 2010 Australia

**Keywords:** Epitranscriptome, RNA methylation, 5-Methylcytosine, NSUN2, TRDMT1, Bisulfite conversion, Bisulfite RNA-seq, RNA stability, mRNA translation, Polysome

## Abstract

**Background:**

5-Methylcytosine (m^5^C) is a prevalent base modification in tRNA and rRNA but it also occurs more broadly in the transcriptome, including in mRNA, where it serves incompletely understood molecular functions. In pursuit of potential links of m^5^C with mRNA translation, we performed polysome profiling of human HeLa cell lysates and subjected RNA from resultant fractions to efficient bisulfite conversion followed by RNA sequencing (bsRNA-seq). Bioinformatic filters for rigorous site calling were devised to reduce technical noise.

**Results:**

We obtained ~ 1000 candidate m^5^C sites in the wider transcriptome, most of which were found in mRNA. Multiple novel sites were validated by amplicon-specific bsRNA-seq in independent samples of either human HeLa, LNCaP and PrEC cells. Furthermore, RNAi-mediated depletion of either the NSUN2 or TRDMT1 m^5^C:RNA methyltransferases showed a clear dependence on NSUN2 for the majority of tested sites in both mRNAs and noncoding RNAs. Candidate m^5^C sites in mRNAs are enriched in 5′UTRs and near start codons and are embedded in a local context reminiscent of the NSUN2-dependent m^5^C sites found in the variable loop of tRNA. Analysing mRNA sites across the polysome profile revealed that modification levels, at bulk and for many individual sites, were inversely correlated with ribosome association.

**Conclusions:**

Our findings emphasise the major role of NSUN2 in placing the m^5^C mark transcriptome-wide. We further present evidence that substantiates a functional interdependence of cytosine methylation level with mRNA translation. Additionally, we identify several compelling candidate sites for future mechanistic analysis.

**Supplementary information:**

**Supplementary information** accompanies this paper at 10.1186/s12915-020-00769-5.

## Background

Cells across all domains of life have an impressive ability to ‘decorate’ their RNAs post-transcriptionally; the MODOMICS database [1] currently lists over 170 known types of chemically modified ribonucleosides and over 360 different proteins involved in RNA modification. This chemical diversity abounds among the noncoding (nc) RNAs involved in translation, and the transfer (t) RNA research field in particular has been a principal source of RNA modification discovery for decades [2]. By contrast, despite early indications [3–5], technological barriers hindered research into the presence and specific distribution of modified nucleosides within messenger (m)RNAs and other ncRNAs. This changed when next-generation sequencing methods were adapted, first to detect RNA editing events, reviewed in [6], and soon after to map 5-methylcytosine (m^5^C) and *N*6-methyladenosine (m^6^A) in a transcriptome-wide fashion [7–9].

Expansion in scope and refinement of such methods [10–12] have now produced maps of several modifications in tissues and cells of diverse origins [13–17], spawning the term ‘epitranscriptomics’ to mainly (but not exclusively) refer to research into the function of modified nucleosides in mRNA [18]. Challenges exist not only in the accurate detection of these generally sparse modifications but also in ascribing molecular, cellular and organismic functions to these ‘epitranscriptomic marks’—in mRNA [19–21] as well as in ncRNA [22, 23]. Here, in analogy to DNA epigenetics, the concept of RNA modification ‘writers, readers, and erasers’ has become an influential, if not always perfectly suited, guide to thinking in the field [24–28].

How this plays out can be seen with the long suspected [29] and recently substantiated mRNA destabilising effects of m^6^A. The modification is added co-transcriptionally in the nucleus by the m^6^A ‘writer’ complex, which includes the methyltransferase (MTase) METTL3 (methyltransferase-like protein 3) [30], while it can also be ‘erased’ again by the demethylase ALKBH5 (AlkB homologue 5) [31]. Several proteins have been shown to bind or ‘read’ m^6^A, including members of the YTH domain-containing family (YTHDF). Among them, YTHDF2 is known to promote mRNA decay in the cytoplasm [32, 33]. However, in addition to its role in turnover, m^6^A has also been implicated in mRNA processing, export and translation, reviewed in [28], as well as in editing [34]. Thus, m^6^A illustrates what can be expected of RNA modifications more broadly, namely that they might have diverse, context-dependent functions. Context relates to both, where the modification is found within an RNA (e.g. sequence, structure and modification level; proximity or overlap with other functional/regulatory RNA features) but also the broader cellular milieu (e.g. availability of ‘readers’ and their downstream effectors) [21, 27].

m^5^C is present in multiple tRNAs, where it can influence the accuracy of translation and tRNA stability [35], thereby also affecting the formation of tRNA-derived small regulatory ncRNAs [36–38]. m^5^C sites are also found in ribosomal (r)RNA, and they can affect ribosome biogenesis, stability and translational performance [39]. The eukaryotic m^5^C ‘writers’ are the seven members of the NOL1/NOP2/SUN domain (NSUN) MTase family and TRDMT1 (tRNA aspartic acid MTase 1; *a.k.a.* DNA MTase homologue 2, DNMT2). They have mostly been characterised as either targeting tRNA (NSUN2, 3, and 6; TRDMT1) or rRNA (NSUN1, 4, and 5) [21, 40]. Despite their seemingly ‘housekeeping’ functions, these MTases display complex expression patterns during development and disease, especially in cancer, and mutations in several of them cause human genetic disease [40–43]. One explanation for their complex biology might be that these MTases modify additional substrates outside of the tRNA and rRNA realm. Indeed, NSUN7 was recently identified as a modifier of enhancer RNAs [44], but NSUN2 has also repeatedly been found to methylate sites outside of its purview [9, 45–50]. Two ‘readers’ of m^5^C have been reported, the mRNA export adapter ALYREF (Aly/REF export factor) [49] and the DNA/RNA-binding protein YBX1 (Y-box binding protein 1) [47, 51], implying certain molecular functions (see below). Finally, a potential route to ‘erase’ m^5^C from RNA is indicated by the presence of 5-hydroxymethylcytosine (hm^5^C), 5-formylcytosine and 5-carboxylcytosine in RNA, which represent intermediates in an oxidative demethylation pathway initiated by ten-eleven translocation (TET) dioxygenases [52–55].

Transcriptome-wide m^5^C maps at variable depth are by now available for several tissues and cell lines of human/mouse [9, 37, 45–50, 56–59], zebrafish [51], plant [60–62], archaeal [63], and even viral [64, 65] origin, persistently identifying sites with biased distribution in mRNAs and/or ncRNAs, reviewed in [66]. Several studies have further suggested regulatory roles for m^5^C in mRNAs. For example, it was shown in the context of leukaemia that m^5^C in nascent RNA mediates formation of specific active chromatin structures [67]. m^5^C can also guide systemic mRNA transport in plants [62], promote nuclear export of mammalian mRNA in conjunction with ALYREF [49] and enhance mammalian and zebrafish mRNA stability facilitated by YBX1 [47, 51]. Further, a negative correlation was noted between translation of mammalian mRNAs and the presence of m^5^C sites transcriptome-wide [48]. Finally, there is a body of work on individual mRNAs and their regulation by m^5^C at the levels of stability and translation in the context of cell proliferation and senescence [68, 69].

Different approaches based on high-throughput RNA-seq as a readout have been developed to map m^5^C. One is to perform an immunoprecipitation of cellular RNA fragments with anti-m^5^C antibodies (m^5^C-RIP) [60, 63]. Other approaches use enzyme-trapping, either by over-expressing a mutant MTase that cannot resolve the covalent enzyme-RNA intermediate (methylation iCLIP or miCLIP) [45], or by prior incorporation of 5-azacytidine into cellular RNA, which then covalently traps endogenous MTases to their substrates (Aza-IP) [46]. Further, in analogy to epigenetic detection in DNA, resistance of m^5^C to conversion into uridine by bisulfite treatment of RNA has also been used (bisulfite RNA-seq, bsRNA-seq) [9, 48, 49, 51, 56, 58, 61]. Imperfections of each method have been noted, for example, in m^5^C-RIP, antibody specificity is crucial, while for miCLIP and Aza-IP, sensitivity might not reach lower abundance targets. All this can account for inconsistencies in site detection across different methods [10, 66]. bsRNA-seq is not completely specific to m^5^C (e.g. it also detects hm^5^C) and is affected by incomplete conversion of unmodified cytosines due to RNA structure and the variability in reaction conditions. Stringency criteria to balance false negative against false positive site calls have also been set differently between studies, leading to, for example, drastically different estimates for sites in mRNA from a handful to thousands [10, 58]. Regardless of the chosen method, a redeeming feature might be that with greater insight into experimental limitations and with refinement of bioinformatic methods, new, better quality m^5^C epitranscriptomic maps are now superseding early, pioneering attempts.

Here, we pursued the molecular roles of m^5^C in human mRNA with an emphasis on any link to translation. RNA isolated from multiple polysome profiling fractions was subjected to efficient bisulfite conversion followed by RNA sequencing (bsRNA-seq), rigorous candidate m^5^C site calling and validation. Bioinformatic analyses identified the sequence and structural context of sites, their preferred location along mRNA, and multiple correlative links to translation.

## Results

### Transcriptome-wide bsRNA-seq after separation by translation state

**Fig. 1:**
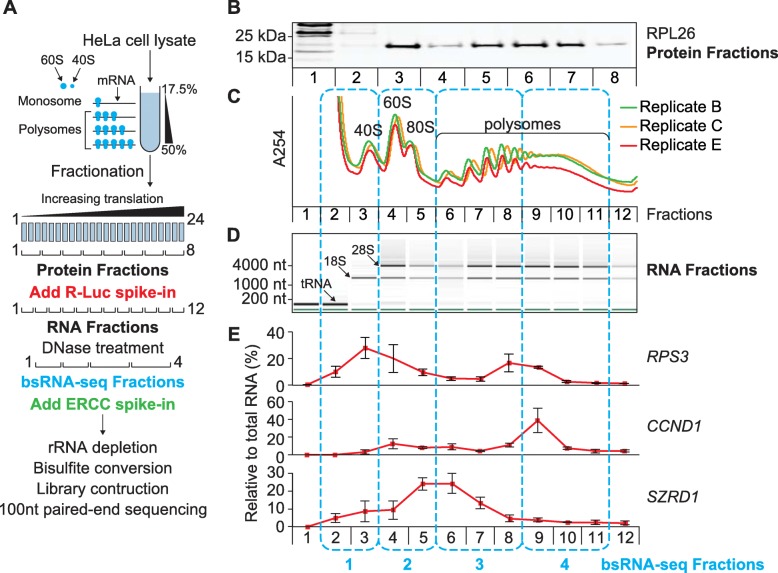
Workflow of polysome profiling and sample selection for bisulfite (bs)RNA-seq. HeLa cell lysates were separated by ultracentrifugation through linear sucrose density gradients. Twenty-four fractions per gradient were taken and combined for subsequent analyses as indicated. The four pooled fractions chosen for bsRNA-seq are indicated by blue boxes. **a** Principle of using sucrose density gradient ultracentrifugation to separate mRNAs by ribosome association (top) and scheme for merging the 24 fractions into different pools for downstream analyses (bottom). First, subsamples were taken and three adjacent fractions were merged to generate eight samples for Western blotting (*Protein Fractions*). Second, fractions were spiked with a Renilla luciferase (*R-Luc*) in vitro transcript and combined pairwise to generate 12 merged fractions (*RNA Fractions*). Total RNA was isolated, DNase treated, assessed for integrity and used for RT-qPCR. Third, *bsRNA-seq Fraction* pools were created as follows: (pool 1: RNA Fractions 2–3; pool 2: 4–5; pool 3: 6–8; pool 4: 9–11). 10 μg of total RNA from each pool was spiked with the ERCC in vitro transcripts, rRNA depleted and sodium bisulfite treated prior to library construction and high-throughput Illumina sequencing. **b** Distribution of Ribosomal Protein L26 (RPL26) across gradients. Protein fractions were subjected to western blotting (equal proportions were loaded). Replicate B is shown as an exemplar of all biological replicates. **c** Absorbance traces (254 nm) across the three biological replicate gradients processed for bsRNA-seq. **d** Distribution of tRNA and rRNA across gradients. RNA fractions were analysed by microfluidic electrophoresis (equal proportions were loaded). The pseudo-gel image for replicate E is shown (see Fig. S1A for data from all replicates). **e** Distribution of representative mRNAs across gradients were determined by RT-qPCR. Results for three mRNAs of different coding region length are shown: *RPS3* (ribosomal protein S3), *CCND1* (cyclin D1) and *SZRD1* (SUZ RNA binding domain containing 1) (see Fig. S1B for further examples). mRNA levels were normalised to *R-Luc*, rescaled as percentage of total signal, and are shown as mean with error bars indicating ± standard deviation

For polysome profiling, rapidly growing HeLa human cervical cancer cells (in biological triplicates B, C and E) were lysed in the presence of cycloheximide, lysates separated by ultracentrifugation through linear sucrose density gradients and multiple fractions taken [70] (Fig. 1a and Table S1 for parameters of each lysate). To monitor efficacy of separation on the gradients and reproducibility across replicates, the absorbance profile at 254 nm was recorded and the distribution of Ribosomal Protein L26 (RPL26) measured (Fig. 1b,c). RNA was isolated from fractions, which were spiked with Renilla Luciferase (*R-Luc*) RNA transcribed in vitro (sequences shown in Table S2), and its integrity checked (Figs. 1d and S1A). RNA fractions were then used in RT-qPCR (primers listed in Table S3) to establish the sedimentation behaviour of multiple cellular mRNAs (Figs. 1e and S1B). To best capture the varying mRNA profiles and, therefore, different translation states, we pooled RNA samples into four final fractions for bsRNA-seq. Fraction 1 encompassed the small ribosomal subunit peak, fraction 2 included the large ribosomal subunit and monosomal peaks, whereas fractions 3 and 4 covered light and heavy polysomal peak regions, respectively (indicated by the blue boxes in Fig. 1). Pooled bsRNA-seq fractions were prepared from each biological triplicate (Fig. S1C), laced with the ERCC (External RNA Controls Consortium) spike-in mix [71], depleted of rRNA and subjected to bisulfite conversion (see Fig. S1D for RNA integrity analyses before and after these steps). Libraries (termed LibB1–4, LibC1–4, and LibE1–4) were prepared and subjected to Illumina HiSeq sequencing (bsRNA-seq).

### Transcriptome-wide identification of candidate m^5^C sites

bsRNA-seq yielded on average ~ 55 million (M) read pairs per library after initial processing. Reads were mapped to ‘bisulfite-converted’ references as shown in Fig. S2 and detailed in “Methods”. Of further note, the human reference genome was combined with the spike-in sequences, whereas dedicated references were used for rRNA and tRNA mapping. On average, ~ 58% of reads were uniquely mapped to the genome (an additional ~ 1.4%, ~ 0.07%, ~ 4.9%, and ~ 0.04% of reads mapped to the ERCC and *R-Luc* spike-ins, rRNA, and tRNAs, respectively; mapping statistics are given in Table S4).

**Fig. 2:**
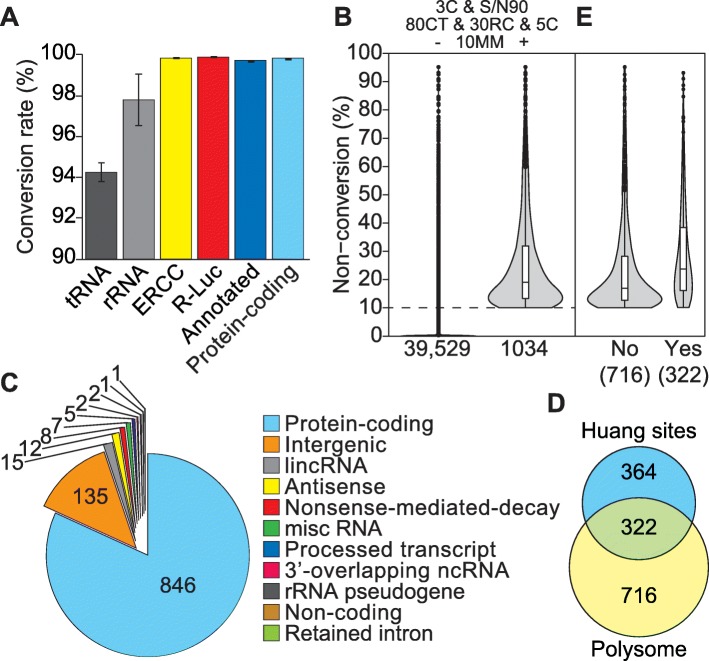
bsRNA-seq performance and transcriptome-wide candidate m^5^C site distribution. bsRNA-seq fraction library mapping data were combined into one composite dataset for each biological replicate (e.g. LibB1–4 yielded cLibB, see Fig. S2). **a** Cytosine conversion rate within different RNA types based on initial read mapping. tRNA and rRNA data are based on dedicated mapping to specialised references, others (ERCC and *R-Luc* spike-ins, all annotated transcripts and protein-coding transcripts [based on GENCODE v28]) are from mapping to the general reference genome (see ‘Methods’ for details). Shown are mean values across the three biological replicates with error bars indicating ± standard deviation. **b** Violin plots showing cytosine non-conversion range of transcriptome-wide m^5^C candidate sites, present in all three replicates and after 3C & S/N90, 80CT, 30RC and 5C filtering, either before (left) or after application of a ≥ 10% average non-conversion cut-off (10MM, right; 1034 sites pass all filters, see main text for details). **c** Distribution of 1034 transcriptome-wide m^5^C candidate sites across RNA biotypes. Site annotation was according to the longest transcript variant recorded in GENCODE v28. Note that 84 of the 135 ‘intergenic’ sites are in fact annotated as tRNAs in RefSeq. **d** Venn diagram showing overlap between 1034 m^5^C candidate sites identified here and a set of 686 sites reported for poly-A-enriched HeLa cell RNA elsewhere [48]. Huang et al. applied bsRNA-seq to HeLa control, NSUN2 knock-out and NSUN2-rescue samples. A union of called sites with at least 10% non-conversion in any of their three samples were used for this analysis. **e** Violin plots showing cytosine non-conversion range of m^5^C candidate sites reported here after subdivision into those that do (Yes) or do not (No) overlap with the set reported by [48]

Next, we merged the four bsRNA-seq fraction libraries into one composite library per biological replicate (termed **cLibB**, **cLibC** and **cLibE**, i.e. *N* = 3) to approximate a total cellular RNA analysis (Fig. S2). Using the initial read mapping, we then assessed overall cytosine conversion for different RNA types. We saw near complete conversion (> 99.8%) of both ERCC and *R-Luc* spike-ins, which are devoid of modified nucleosides and thus attest to the efficiency of the bisulfite conversion reaction (Fig. 2a). C-to-T conversion was also very high across the transcriptome (~ 99.7% for all annotated RNAs; ~ 99.8% for transcripts of protein-coding genes), consistent with a rare occurrence of m^5^C sites in mRNA [47–50, 58]. Conversion levels for tRNAs were lower (~ 94.2%), as expected from the common presence of m^5^C sites in tRNAs. The conversion of rRNAs was intermediate (~ 97.8%), which is inconsistent with the sparseness of m^5^C sites within mature rRNAs (only two sites known in 28S rRNA). We further visually inspected non-conversion at individual cytosine positions for multiple spike-in RNAs and tRNAs as well as for 28S, 18S, and 5.8 rRNAs (Fig. S3). Initial read mapping already reported high levels of non-conversion at known m^5^C positions within tRNAs as well as near complete conversion at other tRNA positions and at all cytosines within the spike-in transcripts (top panels in Fig. S3A,B). By contrast, mapping to rRNAs suffered from incomplete cytosine conversion in multiple clusters (top panels in Fig. S3C), particularly in 28S rRNA. This clustered non-conversion likely reflects the known sensitivity of the bisulfite reaction to secondary structure [72–74] and indicated additional filtering as necessary for high-confidence site calls.

As a first step towards that, we removed reads that contain more than three non-converted cytosines from the initial read mapping (the ‘3C’ filter) [37, 48, 63]. While this did not affect the analysis of spike-in RNAs and most tRNAs (middle panels in Fig. S3A,B), it noticeably reduced, but did not completely eliminate, clustered non-conversion for rRNAs (middle panels in Fig. S3C). Thus, as also suggested by others [48], we further implemented a ‘signal-to-noise’ filter that suppresses site calls at positions where less than 90% of mapped reads passed the 3C filter (‘S/N90’). Combined application of the 3C and S/N90 filters (3C & S/N90) did not change site calls for most tRNAs, although predictably, those with more than three genuine m^5^C sites were affected (bottom panels in Fig. S3B). While detection of the two known m^5^C sites at positions 3761 and 4417 in 28S rRNA benefited from the 3C filter, they were both flagged as unreliable by the S/N90 filter (compare middle and bottom panels in Fig. S3C, as well as the ‘zoomed plots’, Table S5A). On balance, we accepted this tendency for false negative site calls, at least in tRNA and rRNA, in favour of suppressing false positive calls transcriptome-wide.

Additional criteria we implemented for inclusion as a candidate m^5^C site were as follows: coverage above 30 reads (‘30RC’), at least 80% of bases identified are cytosine or thymidine (‘80CT’) and a minimal depth of at least five cytosines (‘5C’). Reproducibility of sites called in this way was high across the biological triplicates (*R*^2^ > 0.95; Fig. S4A). Nevertheless, most called sites had very low cytosine non-conversion (e.g. ≤ 1% on average for 35,090 of a total 39,529; Fig. 2b). Thus, to select sites likely to be ‘biologically meaningful’, we finally required an average non-conversion level across replicates of at least 10% (‘10MM’). This identified 1034 high-confidence candidate m^5^C sites in the transcriptome-wide mapping (Fig. 2b, Table S5B), with only a minority of sites showing very high levels of non-conversion (e.g. ~ 5% had levels above 60%). A total of 322 of these 1034 sites were also included in a set of HeLa cell m^5^C sites reported recently based on bsRNA-seq with similar mapping and site selection strategies [48] (Fig. 2d). The underlying concordance of the datasets is likely higher, as our data has around fourfold greater depth of coverage and overlapping the two site lists further suffers from non-conversion thresholding effects (leading to an exaggerated lack of overlap between sets around the 10% cut-off; Fig. 2e). Given their critical impact on data clean-up, the specific effects of the 3C and S/N90 filters on numbers of called sites in different RNA types are shown in Fig. S4B (with all other filters left in place). Notably, the great majority of candidate m^5^C sites (846 of 1034) was found in transcripts of protein coding genes (Fig. 2C); these became the main focus of further analyses, as detailed below.

### Candidate m^5^C sites in tRNAs and other ncRNAs

Given that polysomal RNA was our source material, coverage for most ncRNAs was not expected to be high. Nevertheless, ~ 18% of sites that passed all our criteria mapped to ncRNA biotypes (Fig. 2c). Notable numbers were found in long intergenic ncRNAs (15 sites) and antisense transcripts (12 sites). These include sites in ribonuclease P RNA component H1 (*RPPH1*; *chr14:20,343,234*), small Cajal body-specific RNA 2 (*SCARNA2*; *chr1:109,100,508*), RNA component of signal recognition particle 7SL1 (*RN7SL1*; *chr14:49,586,869*) and two RN7SL pseudogenes, *RN7SL395P* (*chr8:144,785,379*) and *RN7SL87P* (*chr5:144,140,971*), as well as sites in the two vault RNAs, *vtRNAs1–1* (*chr5:140,711,344* and *chr5:140,711,359*) and *vtRNA1–2* (*chr5:140,718,999*), all of which have been previously reported [9, 46, 75]. Additionally, we identified candidate sites that had not been specifically noted by previous studies, in NSUN5 pseudogene 2 (*NSUN5P2; chr7:72,948,484*), telomerase RNA component (*TERC; chr3:169,764,738*) and nuclear paraspeckle assembly transcript 1 (*NEAT1*; *chr11:65,425,307*). Although it did not fulfil our coverage criteria, we also saw evidence of non-conversion in *SNORD62B* (*chr9:131,490,541*). The sites in *RPPH1*, *SCARNA2*, *NSNU5P2* and *SNORD62B* were further validated in independent biological samples (see below).

A total of 135 sites were located in intergenic regions, according to GENCODE v28 annotation; however, 84 of these reside within tRNAs according to RefSeq annotation. We systematically identified sites in tRNAs from reads that uniquely mapped within the processed tRNA sequence coordinates according to our bespoke pre-tRNA reference (see “Methods”; ~ 175,000 reads per cLib/replicate). tRNA coverage in our data is comparatively low as the bulk of tRNA sediment near the top of the gradient (Fig. 1c), a region we did not include in our bsRNA-seq fraction selection. Nevertheless, we identified a total of 119 candidate m^5^C sites in 19 tRNA iso-decoders (Table S5C). These sites typically show a high cytosine non-conversion level (see examples in Fig. S3C), and they are near exclusively located in the anticipated tRNA secondary structure positions (Fig. S4C). The abundant identification of the major known tRNA sites confirms the reliability of our dataset.

### Validation of candidate m^5^C sites and their NSUN2-dependence

**Fig. 3:**
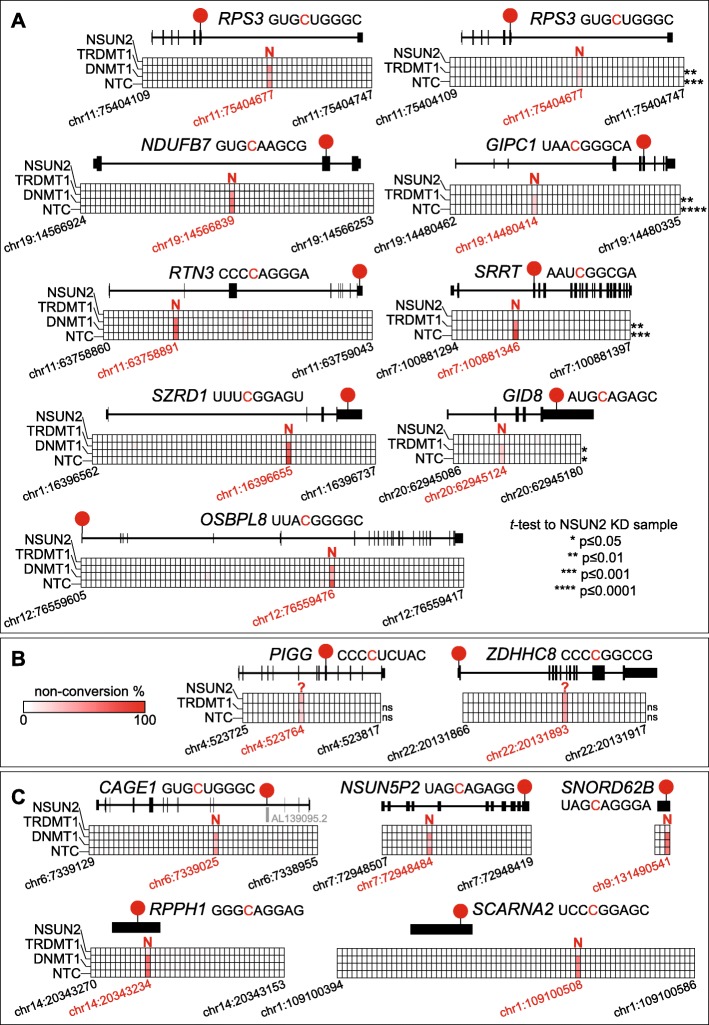
Validation and NSUN2-dependence of candidate m^5^C sites in mRNA and ncRNA. Amplicon-specific bsRNA-seq was performed with total RNA isolated from HeLa cells after siRNA-mediated knockdown targeting NSUN2 or TRDMT1 along with control siRNAs (targeting DNMT1 or a non-targeting control [NTC]; see Fig. S5 for knockdown efficiency; see Table S6 for read coverage details). Grids showing cytosine position along the analysed transcript section (in columns; genomic coordinates for the first and last cytosine, and the candidate m^5^C site are given; knockdown sample indicated on the left) with each square coloured by observed cytosine non-conversion (white-to-red colour scale; shown in panel **b**). The longest transcript isoform (based on Ensembl) is shown above the grid with candidate m^5^C site position (red circle) and sequence context indicated (non-converted cytosine in red). The enzyme identified to mediate methylation is also indicated: N – NSUN2; ? – unknown. ‘Confirmatory’ data (*N* = 1) using NSUN2, TRDMT1, DNMT1 and NTC samples was generated for sites with high coverage. ‘In-depth’ data (*N* = 2 to 3) using NSUN2, TRDMT1 and NTC samples was obtained for lowly covered sites. Asterisks indicate Student’s *t* test *p* value between a given sample and the NSUN2 knockdown. See Fig. S6 for additional candidates. **a** ‘Confirmatory’ data (left panel) for mRNA candidate sites in *RPS3* (ribosomal protein S3), *NDUFB7* (NADH:ubiquinone oxidoreductase subunit B7), *RTN3* (reticulon 3), *SZRD1* (SUZ RNA binding domain containing 1) and *OSBPL8* (oxysterol binding protein-like 8). ‘In-depth’ data (right panel) shows the average for sites in *RPS3*, *GIPC1* (GIPC PDZ domain family member 1), *SRRT* (serrate) and *GID8* (GID complex subunit 8 homologue). **b** ‘In-depth’ data for two mRNA candidate sites (*PIGG* [phosphatidylinositol glycan anchor biosynthesis class G] and *ZDHHC8* [zinc finger DHHC-type containing 8]) that showed no response to either NSUN2 or TRDMT1 knockdown. **c** ‘Confirmatory’ data for ncRNA candidate sites in *CAGE1* (cancer Antigen 1), *NSUN5P2* (NSUN5 pseudogene 2), *SNORD62B* (small nucleolar RNA, C/D box 62B), *RPPH1* (ribonuclease P RNA component H1) and *SCARNA2* (small Cajal body-specific RNA 2)

We employed a targeted approach termed amplicon-bsRNA-seq to confirm the presence of selected sites in independent biological samples. It uses RT-PCR to amplify specific transcript regions after bisulfite conversion and purified amplicons are sequenced using Illumina MiSeq technology. We combined this with siRNA-mediated knockdown of specific RNA methyltransferases (see below) to exclude false positives and further to determine MTase site specificity. Altogether, we report amplicon-bsRNA-seq data for 26 different RNAs in this study, including two *R-Luc* spike-ins and two tRNAs (as controls; Fig. S5) as well as 17 mRNAs (two sites in the 5′UTR, nine in the CDS and six in the 3′UTR) and five ncRNAs (Figs. 3 and S6; see also Table S6). Candidate sites for amplicon-bsRNA-seq were primarily selected based on their non-conversion level. As further described below, we generated two datasets, ‘confirmatory’ and ‘in depth’, which mainly targeted sites with high (> 30%) or low (< 30%) non-conversion level, respectively (Table S6). Further, we considered the expression level of the RNAs containing candidate sites. We chose RNAs with comparatively high expression level (Table S6), although some lowly expressed RNAs were also included out of interest.

New total RNA samples from HeLa cells as well as from two prostate cell lines (epithelial PrEC and cancerous LNCaP) were prepared to test a potential dependence of site presence on the tissue/source material [49, 56]. To test the MTase-dependence of sites, we used siRNA-mediated knockdown (KD) targeting NSUN2 or TRDMT1, alongside controls (siRNA targeting m^5^C:DNA methyltransferase 1 [DNMT1] or a non-targeting control [NTC] siRNA). Knockdown efficiency was monitored for each sample by Western blotting and RT-qPCR (Fig. S5A,B).

Two independent sample sets for amplicon-bsRNA-seq experiments were generated. The ‘confirmatory’ set (*N* = 1) targeted sites with high cytosine non-conversion level in all three cell lines, combining HeLa cells with the full panel of siRNAs, LNCaP cells with siRNAs against NSUN2 and NTC, while PrEC cells were used in non-transfected form only. The ‘in-depth’ set was performed in biological triplicates (*N* = 3) and targeted sites with lower non-conversion in HeLa cells only, combined with knockdown of NSUN2, TRDMT1 and NTC control. In vitro transcribed *R-Luc* spike-in controls were added prior to RNA bisulfite treatment and efficient conversion was observed for all samples (Fig. S5C). Assessment of tRNA^Gly^ (GCC) and tRNA^Thr^ (UGU) sequences generally showed high non-conversion at the known m^5^C positions (TRDMT1 targets C38, NSUN2 targets C48–50) with complete conversion at all other assessed cytosines. Importantly, non-conversion was selectively reduced at C38 in tRNA^Gly^ (GCC) in all TRDMT1 KD conditions, while the variable loop positions C48–50 strongly reacted to NSUN2 KD (Fig. S5D). NTC transfection and DNMT1 KD had no such effects on tRNA non-conversion levels. Using the ‘confirmatory’ HeLa cell sample set, we then confirmed three known NSUN2-dependent sites [9], in the *NAPRT* and *CINP* mRNAs (Fig. S6A, top row) as well as in the ncRNA *RPPH1* (Fig. 3c, bottom left; see figure legends for full gene names). Altogether this established selective MTase KD and efficient bisulfite conversion.

Next, we tested 14 mRNA candidate m^5^C sites in HeLa cells, representing a range of coverage and non-conversion levels as well as various mRNA regions (two sites in the 5′UTR, eight in the CDS and four in the 3′UTR, respectively). Seven sites were validated in the ‘confirmatory’ samples and each showed clear reduction with NSUN2 KD (*RPS3*, *NDUFB7*, *RTN3*, *SZRD1*, *OSBPL8* in Fig. 3a, left column; *SCO1*, *MCFD2* in Fig. S6A, middle row). Eight sites were reproducibly detected in the in-depth samples; four showed clear and statistically significant reduction with NSUN2 KD (*RPS3*, *GIPC1*, *SRRT*, *GID8*; Fig. 3a, right column). Note, that the *RPS3* site was validated with both sample sets, indicating that the ‘confirmatory’ samples are still suitable for candidate evaluation. Two sites still appeared to respond to NSUN2 KD albeit without reaching significance, likely because their low non-conversion level would require more replication (*CCT5*, *NSUN2*; Fig. S6A, bottom row). Interestingly, two sites convincingly lacked responses to either MTase KD (*PIGG*, *ZDHHC8*; Fig. 3b). NSUN2-independence for the *PIGG* site has been noted previously [48], we additionally show its TRDMT1-independence here. Using the ‘confirmatory’ samples, we further confirmed presence and selective sensitivity to NSUN2 KD for four sites in ncRNAs (the *RPS3 pseudogene* (AL139095.2) encoded in the *CAGE1* intron, *NSUN5P2, SNORD62B*, *SCARNA2*; Fig. 3c). Finally, six candidate m^5^C sites were also explored using the two prostate cell lines (four in mRNAs: *SZRD1*, *RTN3*, *SRRT*, *PWP2*, and two in ncRNA: *SCARNA2*, *SNORD62B*; Fig. S6B). All these sites were found in both PrEC and LNCaP cells and they each responded to NSUN2 KD in LNCaP cells.

In summary, the presence of all chosen sites was validated by amplicon-bsRNA-seq, even though based on polysome bsRNA-seq they varied widely in coverage (e.g. *PWP2*, *ZDHHC8*, *SNORD62B*, *NAPRT* were actually below our 30RC cut-off) and in cytosine non-conversion level. Regarding the latter, there was a reasonably good concordance between non-conversion level by transcriptome-wide and amplicon-specific measurements (e.g. *CCT5* 13% vs 5%, *RSP3* 25% vs 13% *SRRT* 63% vs 77%; averages from polysome bsRNA-seq versus NTC in-depth sample, respectively; see Table S6). This highlights the reliability of both, transcriptome-wide and amplicon-bsRNA-seq data. Although only based on a limited comparison, sites in both mRNA and ncRNA could be found in all three cell lines, suggesting at least some overlap in m^5^C profile between different cell types. Importantly, of the 17 mRNA and five ncRNA sites examined by amplicon-bsRNA-seq here, all ncRNA sites and 15 in mRNA were found to be targeted by NSUN2 and none by TRDMT1. Two mRNA sites did not respond to either knockdown and thus might be targeted by other MTases. Altogether, these data further substantiate the notion that NSUN2 has a broad but not exclusive role in modifying cellular transcriptomes [47–50, 57, 58].

### Candidate m^5^C sites display enrichment in multiple mRNA regions

Approximately 82% of sites we identified were present in transcripts of protein-coding genes (Fig. 2c). In support of specific roles, these sites are enriched for several Gene Ontology (GO) pathway terms, particularly those related to cell adhesion, translation and RNA processing/turnover (Fig. S7A, Table S7). These results broadly match findings in similar cell contexts [49, 50].

**Fig. 4:**
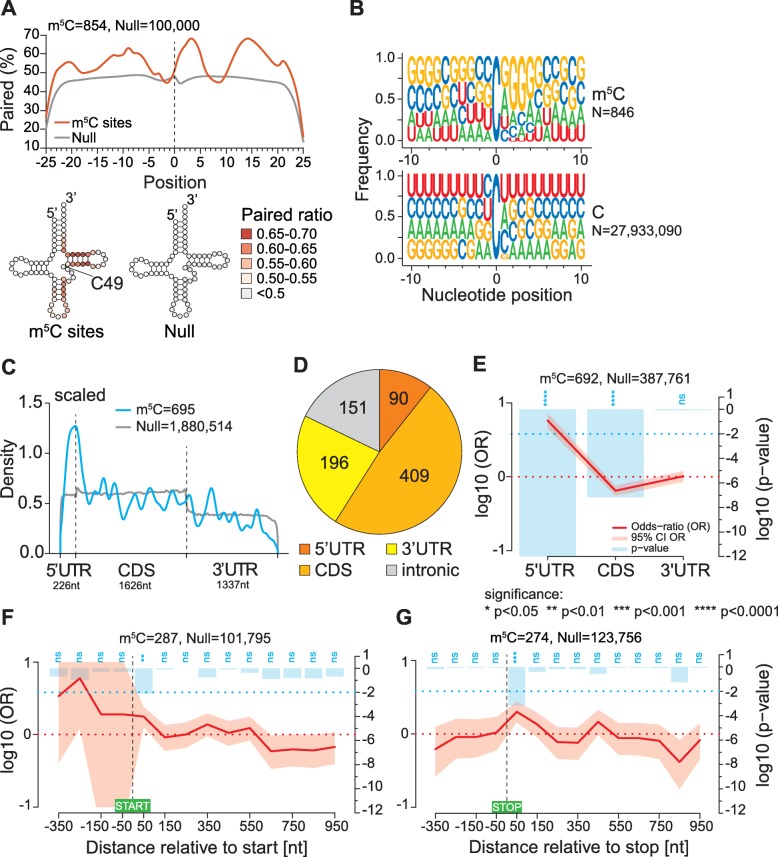
Sequence context and structural characteristics of candidate m^5^C sites. **a** Base-pairing propensity meta-profile of regions surrounding candidate sites within transcripts derived from protein-coding genes. Regions around randomly selected Cs from transcribed genome regions were used as control (top). The base pairing percentage (white-to-red colour scale) of regions ± 20 nt around candidate sites are displayed within a cloverleaf structure, aligning candidate sites with the C49 structural position of tRNA (bottom). **b** Sequence context of candidate sites in transcripts derived from protein-coding genes (top) in comparison to all cytosines in the same transcripts (bottom). Sequence logos for sites separated by different transcript regions are shown in Fig. S7C. **c** Metagene density plot showing distribution of candidate sites within mature mRNAs. Each mRNA region was scaled to its median length, indicated underneath. Candidate site distribution is shown in blue, and background cytosine distribution is shown in grey. **d** Pie chart showing distribution of candidate sites across 5′UTR, CDS, 3′UTR and introns of transcripts from protein-coding genes. **e**–**f** Spatial enrichment analyses of candidate sites within mRNAs. Sites are placed into chosen bins as indicated on the *x*-axis. Site distribution across bins is compared to matching randomised cytosine sampling (Null) and the log10 Odds-ratio (OR) is plotted as a red line with the 95% confidence interval (CI) shaded. Significance of enrichment is plotted as the log10 *p* value by blue bars (legend given in panel **e**). **e** Distribution of candidate sites across the 5′UTR, CDS and 3′UTR of mRNA. **f**, **g** Distribution of candidate sites across the start (**f**) and stop (**g**) codon regions (from − 400 nt to + 1000 nt relative to the first position of respective codon) using a bin width of 100 nt. Note that small discrepancies in expected site numbers between panels are due to inclusion of eight sites from the ‘NMD’ RNA biotype in **a**, and use of different GENCODE annotation versions, i.e. v28 in (**a**–**d**) and v20 in (**e**–**g**)

Given the broad role of NSUN2 in mRNA cytosine methylation, it can be expected that sites share features of the canonical tRNA substrates of the enzyme. Thus, we predicted RNA secondary structure around sites using the RNAfold tool in the ViennaRNA Package 2.0 [76]. Compared to randomised sequences, this shows a relatively lower base-pairing tendency for the region immediately upstream of sites (position − 4 to − 1). Either side of this region there are patterns of alternating short segments with increased or decreased propensity for base-pairing (Fig. 4a, top panel), neatly resembling the context of the major NSUN2-dependent sites in tRNA structural positions C48–50 (Fig. 4a, bottom panel). We also investigated the sequence context around the modified cytosine using ggseqlogo [77]. We noted a moderate bias for C or G in the two upstream positions and a moderate-to-strong G-bias in downstream positions 1–5, yielding a consensus of C/G-C/G-**m**^**5**^**C**-G/A-G-G-G-G (Fig. 4B). Again, this consensus is similar to the C48–50 position in tRNA and its immediate 3′ sequence context. These findings elaborate on earlier reports that ‘non-tRNA’ m^5^C sites reside within CG-rich regions [9, 49, 75]. The similarity to tRNA structure was further noted for sites in vtRNAs [75]. They closely match recent findings that NSUN2-dependent sites in mRNAs reside in a sequence and structural context resembling tRNAs [48].

Next, we analysed candidate m^5^C site distribution along mRNA regions. A scaled metagene analysis showed a marked increase in sites around start codons (Fig. 4c), confirming prior reports in human and mouse [49, 56, 57]. While sites were found in both UTRs and also in intronic regions, just under half of them were located within the CDS (Fig. 4D). CDS sites showed some codon bias, being enriched in eight codons specifying 5 amino acids, primarily in the first and second codon positions (Fig. S7B). Despite the numerical predominance of CDS sites, spatial enrichment analysis of sites in mRNA using RNAModR [78] revealed significant overrepresentation of sites in the 5′UTR, with a minor but significant underrepresentation in the CDS (Fig. 4e). Of note, adherence to the site sequence context established above (Fig. 4B) was strongest in the 5′UTR, with some divergence in the 3′ UTR (consensus: G/C-U/G-**m**^**5**^**C**-A/G-G-G-G-G (Fig. S7C). To further inspect site prevalence near start codons, we divided the surrounding region (− 400 to + 1000 nt) into 100-nt bins and directly tested for enrichment (Fig. 4f). The broad window and relatively coarse bin size were necessary to retain statistical power, given the relatively low site numbers in mRNA. This showed a gradient of decreasing site prevalence in 5′ to 3′ direction, confirming the concentration of sites along 5′UTRs. Within 5′UTRs, the − 201 to − 300 interval showed the highest odds ratio, albeit without reaching significance. As the median length of 5′UTRs represented in our data is 226 nt, this could suggest some concentration of candidate m^5^C sites near the mRNA 5′ end; however, site numbers are too low to ascertain this (Fig. S7D, left panel). The 100-nt region immediately downstream of the start codon showed significant site enrichment, while bins within the body of the CDS (> 600 nt downstream of start codons) showed a continued decrease of site density (Fig. 4f). We extended these analyses to several other mRNA features, which mostly remained inconclusive due to diminishing site numbers in any given region. There was, however, a significant site enrichment within the 100-nt interval immediately downstream of stop codons (Fig. 4g) and in the interval 101–200 nt upstream of mRNA 3′ ends as well (Fig. S7D, right panel). Altogether, the diversity of candidate m^5^C site distribution patterns observed here hint at distinct, context-dependent functional roles for m^5^C.

### Transcriptome-wide anti-correlation between cytosine modification level and mRNA translation efficiency

Links to mRNA translation are suggested by several of the site enrichment patterns described above. This was also emphasised in a recent report, showing a significant negative correlation between candidate m^5^C site-content in the CDS and translation efficiency in the HeLa cell transcriptome [48]. To independently verify this, we obtained HeLa cell ribosome profiling data from several studies [33, 79, 80]. Cumulative distribution of translation efficiency values allowed us to compare mRNAs found by us to contain candidate m^5^C sites with the remaining mRNAs. Irrespective of underlying ribosome profiling data, we then saw a clear and significant tendency for site-containing mRNAs to be less well translated (Fig. S8A). In a similar vein, we also assessed any transcriptome-wide relationship with mRNA stability [33, 79] and found site-containing mRNAs to display a significant trend towards longer half-life (Fig. S8B). Interestingly, this latter observation matches findings reported recently for m^5^C-modified mRNAs in mammals and zebrafish [47, 51].

**Fig. 5:**
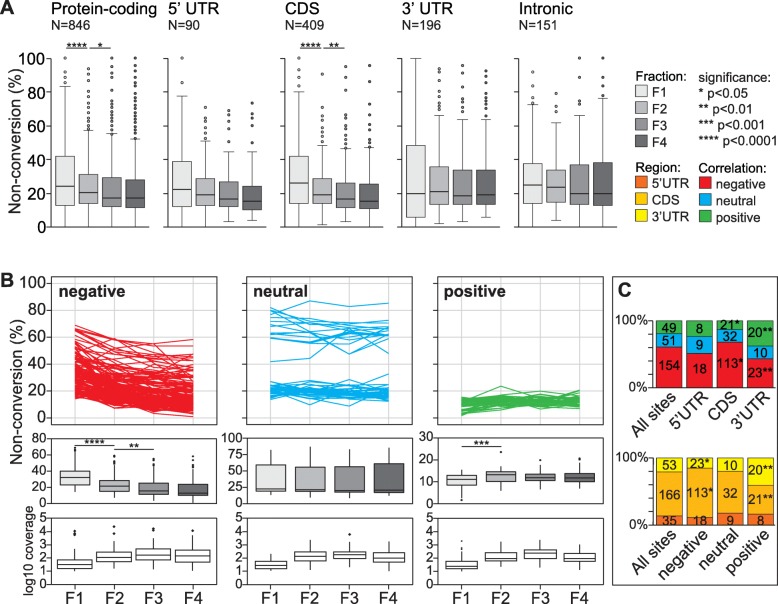
Relationship between non-conversion level at candidate m^5^C sites and mRNA translation state. bsRNA-seq libraries were grouped as biological triplicates per fraction (e.g. LibB1, LibC1 and LibE1 each report on sites detected in bsRNA-seq fraction 1), allowing the calculation of average non-conversion levels per individual site and per fraction. **a** Boxplots showing distribution of candidate site non-conversion levels across the polysome profile. Shown from left to right are all sites in protein-coding RNA (*c.f.* Figures 2c and 4d), as well as subsets of these sites in the 5′ untranslated region (UTR), coding sequence (CDS), 3′UTR and introns. Asterisks indicate Student’s *t* test *p* value comparing adjacent fractions. **b** Non-conversion levels per individual site across the polysome profile were partitioned into nine soft clusters using Mfuzz (see Fig. S9). A total of 254 candidate m^5^C sites in exonic mRNA regions (5′UTR, CDS, 3′UTR designation in panel **a**) were included based on having coverage in at least 9 out of 12 bsRNA-seq fraction samples and ≥ 10 average read coverage in each of the four bsRNA-seq fractions. Mfuzz clusters were grouped into three translation state trend categories by visual inspection, showing a negative (*N* = 154), neutral (*N* = 51) or positive trend (*N* = 49) with polysome association. Top panels: line graphs displaying individual site average non-conversion levels across fractions. Middle panels: boxplots showing distribution of site non-conversion levels in each fraction. Asterisks indicate Student’s *t* test *p* value comparing adjacent fractions. Bottom panels: boxplots showing distribution of site read coverage in each fraction. **c** Stacked bar charts showing distribution of sites in the different translation state trend categories across mRNA regions (top) and distribution of sites in different mRNA regions across translation state trend categories (bottom). Asterisks indicate *p* values following binomial test against the distribution of all sites. The legend given in panel **a** is applicable to all panels. See Fig. S10 for cluster analysis of additional sites with sufficient coverage only in bsRNA-seq fractions 2–4

A unique advantage of our sampling approach is that it allows us to profile the level of cytosine non-conversion at each site across polysome gradient fractions, with bsRNA-seq data for each fraction available in biological triplicates (see Fig. S2, Table S8A). We first assessed the overall non-conversion range of the 846 sites in transcripts from protein-coding genes in each fraction. This showed declining non-conversion levels with increasing ribosome association, with comparisons of fraction 1-to-2 and 2-to-3 reaching statistical significance (Fig. 5a, left panel). This trend was most pronounced with sites in the CDS and still discernible with 5′UTR sites, whereas the 3′UTR and intronic sites did not show a clear trend (Fig. 5a, remaining panels). This demonstrates a negative correlation, on the bulk level, between the extent of cytosine non-conversion and mRNA translation state, primarily driven by observations with CDS sites.

Next, we considered non-conversion levels of sites individually and performed Mfuzz clustering [81]. We selected a set of sites in mature mRNA that had sufficient (≥ 10 reads average) coverage in all four fractions (F1234; 254 sites), as well as a second set that had sufficient coverage in fractions 2–4 but not in fraction 1 (F234; 315 sites). Mfuzz was run requiring 9 clusters (Figs. S9,S10A, Table S8B,C) before re-grouping clusters based on overall non-conversion trends. This generated three major profile patterns for each set, representing positive, neutral and negative correlation with translation state, respectively. Positive and negative pattern sets each showed significant non-conversion level change between fractions as expected (F1234 shown in Fig. 5b; F234 shown in Fig. S10B). Focusing on the set with stronger discriminative potential, F1234, we saw that non-conversion levels were not distributed across patterns equally; most notably, sites with positive patterns typically had low non-conversion levels through the fractions, whereas sites with a neutral pattern were, for unknown reasons, split into two groups, one with ~ 20% and a smaller group with ~ 60% non-conversion (Fig. 5b, top panels).

Notably, site profiles indicating negative correlation were the most common (~ 61%), with positive profiles (~ 19%) being the least frequent (Fig. 5c, top panel). This bias towards negative profiles was moderately but significantly enhanced with CDS sites (~ 68%), whereas 3′UTR sites were significantly underrepresented (~ 43%). Among profiles showing positive correlation with translation, CDS sites were significantly depleted (~ 13%), while 3′UTR sites (~ 38%) were significantly enriched. Conversely, compared to all sites, those in the negative pattern set were moderately but significantly enriched for CDS location and depleted for 3′UTR location. Sites with a positive pattern were depleted for CDS location but enriched for 3′UTR location (Fig. 5c, bottom panel). Many, but not all, of these observations were also made in the less discriminative F234 set (Fig. S10). With the caveat that a proportion of individual site profiles are based on imprecise measurements (see below), the key discernible features from the clustering approach were (a) a preponderance of sites showing negative correlation with translation state and, (b) while 5′UTR sites were relatively unremarkable, there was a tendency for CDS and 3′UTR site to segregate into negative and positive pattern sets, respectively.

### Individual mRNA sites show robust anti-correlation with translation state

To identify individual sites displaying significant non-conversion change across the polysome profile, we performed pairwise logistic regression analysis (Table S8D). In each pairwise comparison, the majority of sites that reached significance showed a negative correlation with translation state; the F1-F2 comparison yielded the largest number of significant sites with the strongest bias towards the negative trend (Fig. S11A). A total of 43% of the sites in the F1234 set but only ~ 7% in the F234 set had significant differences in at least one pairwise comparison (Table S6). Focusing on the F1234 set, 108 sites reached significance comprising a total of 149 significant pair-wise comparisons. Of note, the large majority of these sites represented a negative trend with translation state, and most assignments were based on the F1-F2 and F2-F3 comparisons (Fig. S11B). Sixteen of these sites were in the 5′UTR, 81 in the CDS, and 11 in the 3′UTR, which represents significant enrichment of CDS sites and depletion of 3′UTR sites.

Sites with significant change in cytosine non-conversion level across several fraction steps, or with larger magnitude of change between steps, may represent the most compelling candidates for functional studies. Regarding the former, 12 sites reached significance in all three pairwise comparisons; 10 of these showed a negative trend (17/79 with 14/69 sites showing a negative trend based on two or a single pairwise comparison, respectively). Regarding the latter, 59 of the 149 significant pairwise comparisons satisfied an arbitrarily imposed criterion of ≥ 10% relative non-conversion difference. In total, 54 of these pairs represented a negative step. Furthermore, they were primarily based on the F1-F2 comparison (39; 15 on F2-F3 and 5 on F3-F4). In terms of sites, none of the 12 ‘triple significance’ sites satisfied this criterion at all steps, one having two steps and five having one step of the required magnitude (for the 17 ‘double significance’ sites: three for two steps, 12 for one step; 79 ‘single significance’ sites: 33 for one step). Overall, choosing sites for ‘compelling’ profiles across the polysome gradient primarily, but not exclusively, selects for those showing a negative trend with translation state.

**Fig. 6:**
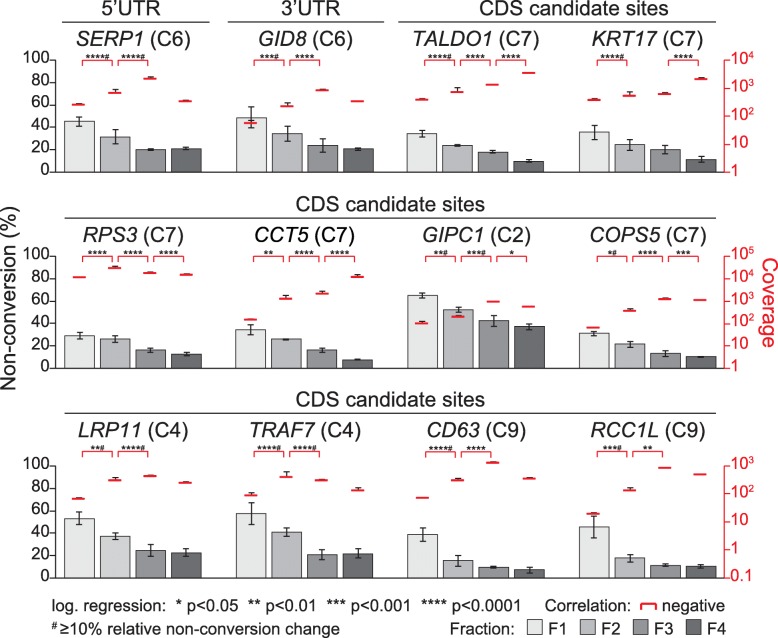
Individual mRNA candidate m^5^C sites showing significant correlation of cytosine non-conversion with translation state. Dual axis charts show cytosine non-conversion (bars) and coverage (red lines) for a given site across the polysome gradient. Data is shown as means across biological triplicates, with error bars indicating standard deviation. Asterisks indicate significance *p* values after logistic regression testing (see key below the charts). The gene name for each candidate site and its position within the mRNA is given. All sites shown here show significant negative non-conversion change in at least two fraction steps and are represented in the F1234 clustering with the cluster number indicated in brackets. mRNAs shown are as follows: *SERP1* (stress associated endoplasmic reticulum protein 1); *GID8* (GID complex subunit 8 homologue); *TALDO1* (transaldolase 1); *KRT17* (keratin 17); *RPS3* (ribosomal protein S3); *CCT5* (chaperonin containing TCP1 subunit 5); *GIPC1* (GIPC PDZ domain containing family member 1); *COPS5* (COP9 signalosome subunit 1); *LRP11* (LDL receptor related protein 11); *TRAF7* (TNF receptor associated factor 7); *CD63* (CD63 molecule); *RCC1L* (RCC1-like)

The profiles of sites selected for significant (for two or more steps) and/or strong change (≥ 10% relative), as well as different level of cytosine non-conversion overall, are shown in Fig. 6 and Fig. S11C,D. The selection further contains several sites that were validated by amplicon-bsRNA-seq. Inspecting the few ‘promising’ sites with positive change showed that several of them actually displayed a complex profile pattern, consistent with their varied membership to the trend patterns described above (Fig. 5b) and leaving even fewer with a clear, monotonously positive association with translation state (Fig. S11D). By contrast, all ‘high-quality’ sites with negative change came from the negative trend pattern (Fig. 5b, left panel) and nearly all displayed a continuous decline in cytosine non-conversion from fraction 1 through to 4 (Figs. 6 and S11C). While a few of these latter sites were situated in the 5′ or 3′UTR, most of them were located in the CDS. Thus, bsRNA-seq has identified candidate m^5^C site in multiple individual mRNAs that suggest an interdependence with translation. These sites/mRNAs are now accessible to functional follow-up studies.

## Discussion

We present here a set of ~ 1000 high-confidence candidate m^5^C sites in the human HeLa cell transcriptome. The great majority of sites were found in mRNAs, and their sequence and structure contexts strongly resembled that of the canonical NSUN2 target region in the variable loop of tRNAs. This matches our extended validations by amplicon-bsRNA-seq, which attributed 21 of 23 confirmed sites to NSUN2. Several findings point towards functional links particularly to translation, including site enrichment in the mRNA 5′ region and near start codons. m^5^C-containing mRNA species further display relatively lower translation efficiency as measured by ribosome profiling. Uniquely, we exploited the generally sub-stochiometric modification level of mRNAs to directly show a prevailing negative correlation between modification state and recruitment into polysomes.

The merits of using bsRNA-seq to discover m^5^C sites transcriptome-wide have been controversially discussed [10, 11, 58, 66]. We contend that the bsRNA-seq approach as presented here is fit-for-purpose as it is based on a combination of efficient bisulfite reaction conditions, bespoke read mapping and conservative site calling from replicate data. Nevertheless, our operationally defined settings to reduce false positives also incur limitations. As illustrated for tRNAs, the 3C criterion biases against detection of more than three closely spaced sites. The ‘S/N90’ criterion hinders detection of true sites within strong RNA secondary structure, as shown for rRNA. Still, these settings are justifiable given that our focus was on sites elsewhere in the transcriptome. Similarly, applying the ‘10MM’ criterion to remove sites with low non-conversion level seems prudent to focus on functionally important sites. However, the use of thresholded data would be problematic for comparative studies of methylation level between tissues or treatment conditions. It may also be unnecessarily stringent when using the data for other purposes, for example when characterising MTase substrate requirements. Supporting the overall validity of our approach is our high amplicon-based validation rate and the extensive site overlap with a recent study that converged on a similarly stringent approach to analyse bsRNA-seq data [48].

Short of an unexpected discovery of novel RNA:m^5^C MTases, sites in the eukaryotic transcriptome at large need to be deposited by one of the existing NSUN enzymes or TRDMT1. The well-characterised NSUN2 has long been the prime suspect, and there is indeed cumulative evidence pointing to NSUN2 modifying both, mRNA and ncRNA from earlier studies [9, 45, 46, 48–50, 57]. We examined this here by amplicon-bsRNA-seq and found 5/5 ncRNA sites and 15/17 mRNA sites tested to be targeted by NSUN2. None was targeted by TRDMT1, leaving two mRNA sites that might be targeted by other MTases. This independently confirms, and adds to, earlier targeted analyses of this kind. For example, the existence and NSUN2-dependence of sites in the ncRNAs *RPPH1*, *SCARNA2* and several vault RNAs has been repeatedly shown [9, 45, 46]. Two of the 15 mRNA sites shown to be NSUN2-dependent here, were reported by us before [9]. Thirteen mouse mRNA sites discovered by bsRNA-seq were independently validated by m^5^C-RIP-seq, although their NSUN2-dependence was not assessed [56]. Further, we and others [48] have found that, transcriptome-wide, m^5^C site context resembles that of the major NSUN2-dependent sites in tRNA structural positions C48–50. Thus, NSUN2 appears to identify all of its targets by recognising tRNA-like features. Curiously, while in tRNA two or three adjacent cytosines are often modified by NSUN2, this seems to be rare in its non-canonical targets. It was also found that most, but not all, transcriptome-wide sites lacked methylation in NSUN2 knock-out cells; those that were NSUN2-independent were situated in a distinct sequence/structural context [48]. Related to that, it was very recently reported that a subset of human mRNA m^5^C sites showed differential cytosine modification levels in TRDMT1 knockdown cells; one site was independently validated [82]. Altogether, multiple lines of evidence implicate NSUN2 as a major, but likely not the only, MTase targeting mRNAs.

Available information on epitranscriptomic marks suggests a range of context-dependent functions and m^5^C is likely no exception. Several observations in this study individually, but not necessarily coherently, suggest functional links to mRNA translation but also stability, broadly concurring with prior evidence. Drawing on published mRNA half-life and ribosome profiling data we saw that, compared to the rest of the transcriptome, m^5^C-containing mRNAs were more stable but less-well translated. A negative transcriptome-wide correlation between m^5^C site presence in human mRNAs and mRNA translation efficiency, as measured by ribosome profiling, was shown before [48] as was a positive transcriptome-wide association with mammalian and zebrafish mRNA stability [47, 51]. Further, effects of m^5^C sites on stability and translation of several individual mRNAs in the context of cell senescence have been reported [68, 69]. Given that these are opposing trends in terms of gene expression output, a reasonable guess would be that different subsets of sites drive each association. Uniquely, by comparing polysome association of modified with unmodified mRNAs of the same type, we directly showed anti-correlation between cytosine modification level and translation efficiency. This was true for most, but not all mRNAs available for this analysis, again suggesting diversity of site functionality. Distribution of candidate m^5^C sites was also uneven in mRNAs. We saw a gradient of increasing site prevalence towards the mRNA 5′ end, resulting in an enrichment in the 5′UTR. Overall, CDS sites showed enrichment in the first and second positions of eight codons. We could also discern site enrichment just downstream of start and stop codons as well as upstream of the mRNA 3′ end. Enrichment of m^5^C sites around the start codon region has been noted repeatedly [47, 49, 56, 57]. Site positioning could be a key determinant of downstream function, and thus might co-segregate with other patterns suggesting distinct functionality. Unfortunately, for the most part, we lacked sufficient site numbers to conduct meaningful statistical analyses of this kind.

Considering the strongest pattern we saw, a negative correlation of m^5^C level with translation, which was strongest with sites situated in the CDS, it is possible to speculate about the underlying mechanism(s). One set of possibilities involves the action of a specific m^5^C reader protein, such as YBX1, already a known regulator of mRNA stability and translation [47, 51]. The reader bound to the CDS could function through steric hindrance, impeding the progression of the elongating ribosome. Given that we inferred translational repression from a lower density of ribosomes along mRNAs, this would require the roadblock to be positioned reasonably early within the CDS, consistent with the observed enrichment of m^5^C sites near the start codon. Alternatively, the reader could interfere with ribosome loading to the mRNA during translation initiation, for example through targeting the role of the cap structure or poly(A) tail in this process. Indeed, YBX1 has been reported to interact with poly(A) binding proteins [47, 51]. Finally, a potential role of m^5^C within the CDS in modulating codon-anticodon interactions, analogous to modifications within the tRNA anticodon loop [83], must also be taken into account. Another contribution of this study is that we provide multiple individual mRNA examples that can be used to distinguish between these possibilities in future work.

## Conclusions

Our findings emphasise the major role of NSUN2 as a major writer enzyme of the m^5^C epitranscriptomic mark transcriptome-wide. We present compelling evidence for a functional interdependence of mRNA cytosine methylation and mRNA translation, indicating that m^5^C may commonly play a negative role in the translational control of mRNAs. However, demonstrating and mechanistically characterising specific causal links between m^5^C and mRNA translation will require in-depth studies of prototypical examples.

## Methods

### Cell growth and maintenance

HeLa (human cervical cancer) cells were obtained from ATCC and grown in DMEM medium (Life Technologies) supplemented with 5% FBS and 2 mM l-glutamine and passaged when 70–100% confluent. Prostate cell lines were obtained from ATCC. PrEC cells (Prostate Epithelial Cells) were cultured in PrEBM basal medium supplemented with PrEGM SingleQuots™ Supplements and Growth Factors (Lonza) and passaged when 80–90% confluent. LNCaP cells (prostate cancer cell) were cultured in T-Medium (Life Technologies) supplemented with 10% FBS and 2 mM l-glutamine and passaged when 90–100% confluent. All cells were incubated at 37 °C with 5% CO_2_.

### Generation of the R-Luc spike-in control RNA sequences

Humanised Renilla Luciferase (*R-Luc*) RNA, transcribed in vitro from the *R-Luc* insert located in pCl-Neo [84], was used as a bsRNA-seq spike-in negative control to assess conversion efficiency. Either this RNA or a second non-humanised *R-Luc* RNA, transcribed in vitro from the *R-Luc* insert in the pRL-TK vector, was used as negative control in the amplicon-bsRNA-seq experiments. RNAs were transcribed using the MEGAscript® T7 Kit (Life Technologies) according to the manufacturer’s protocol. Template DNA was removed using TURBO™ DNase (Ambion) and the RNA cleaned by phenol/chloroform extraction and precipitated. A second DNase treatment step was performed to remove any residual template DNA. The size and integrity of each in vitro transcript was assessed using the RNA 6000 Nano Kit on the 2100 Bioanalyzer (Agilent Technologies).

### Western blotting

For Western blotting of sucrose gradient fractions, the protein fraction samples were used directly for gel electrophoresis without any further protein purification. For Western blotting of methyltransferase knockdown samples, protein was isolated from cells using 300 μl CytoBuster™ Protein Extraction Reagent (Novagen) per well according to the manufacturer’s instructions. Western blotting was performed as standard, separating proteins on NuPage™ 4–12% Bis-Tris Protein Gels (Invitrogen) followed by transfer onto nitrocellulose or PVDF (for IR-Dye detection) membrane. The membrane was blocked in 5% milk in PBS-T (0.05% Tween-20) or Odyssey Blocking Buffer (for IR-Dye detection; LI-COR 927-40000) and probed with primary antibodies: anti-RPL26 antibody (1:1000; Sigma R0780), anti-NSUN2 (1:1000–1:5000; Proteintech 20854-1-AP), anti-TRDMT1 (1:1000; Proteintech 19221-1-AP), anti-tubulin (1:1000; Sigma T6199) or anti-GAPDH (1:1000; Abcam ab9484) at room temperature. The membranes were probed with a secondary antibody: either anti-rabbit-HRP (1:5000; Merck Millipore AP132P), anti-mouse-HRP (1:5000; Agilent Dako P0260), anti-mouse-IR-Dye800 (1:10,000; LI-COR 926-32210) or anti-rabbit-IR-Dye680 (1:10,000; LI-COR 925-68071). For HRP detection, membranes were incubated with substrate (Pierce) and visualised. For IR-Dye detection, the membranes were scanned using the Odyssey® CLx Imaging System (LI-COR).

#### Amplicon bisulfite RNA sequencing data generation and analysis

##### siRNA-mediated knockdown of methyltransferase genes

Gene knockdown was performed by Lipofectamine® RNAiMax (Invitrogen) transfection with SMARTpool siGENOME siRNAs (Dharmacon) targeting *NSUN2*, *TRDMT1*, *DNMT1* and a non-targeting control (NTC) as described previously [9]. Briefly, 1.5 × 10^5^ cells were transfected with 60 pmol siRNAs in a six-well plate format, passaged after 3 days, transfected again and harvested 6 days post initial transfection. To assess knockdown efficiency, protein and RNA were isolated for Western blot (as described above) and reverse transcription followed by quantitative PCR (RT-qPCR) analyses, respectively. Of note, NSUN2 and NTC siRNA transfection of prostate cells was only performed in LNCaP cells as PrEC cells showed strongly decreased viability in response to transfection.

##### RNA extraction and reverse transcription-quantitative PCR (RT-qPCR)

RNA was extracted with TRIzol (Invitrogen) or TRI Reagent® [85] according to the manufacturer’s instructions. Briefly, cells were suspended in 1 ml TRI Reagent per well, mixed with 200 μl chloroform and incubated for 3 min. Phases were separated by centrifugation at 12,000 rpm for 15 min at 4 °C. RNA was precipitated from the aqueous phase for 10 min with 500 μl isopropanol in the presence of 5 μl glycogen (10 mg/ml). RNA was collected by centrifugation at 12,000 rpm for 10 min at 4 °C. The pellet was washed with 75% ethanol, suspended in nuclease-free water and ethanol precipitated overnight at − 20 °C. Precipitates were collected by centrifugation at 12,000 rpm for 15 min at 4 °C and the pellets washed with 75% ethanol and resuspended in nuclease-free water. RNA samples were analysed for quality using a Thermo Scientific™ NanoDrop™ One spectrophotometer (Thermo Fisher) and for integrity using the RNA 6000 Nano Kit on the 2100 Bioanalyzer (Agilent Technologies). RNA was treated using the TURBO DNA-*free*™ Kit (Invitrogen) according to the manufacturer’s instructions, purified by phenol/chloroform extraction and precipitated. An RNA subsample was reverse-transcribed using SuperScript™ III Reverse Transcriptase (Invitrogen) with oligo-dT primers and qPCR performed using Fast SYBR® Green Master Mix (Applied Biosystems). Primer sequences are listed in Table S2. Amplifications were performed in technical triplicates in a 384-well format using the QuantStudio™ 12 K Flex system (Life Technologies). Housekeeping genes (e.g. *GAPDH* or *ACTB* as indicated) were used as internal standard control to normalise expression levels.

##### Bisulfite conversion and amplicon-bsRNA sequencing

The remaining RNA was spiked with 1/1000 (w/w) of *R-Luc* in vitro transcript and 1 μg of RNA subjected to bisulfite conversion as described previously [86]. Purified RNA was reverse-transcribed using SuperScript™ III Reverse Transcriptase (Invitrogen) with random hexamers and subjected to amplicon-specific touchdown PCR. PCR conditions were optimised for each primer set and carried out in technical triplicates (primer sequences specifically target bisulfite converted templates and are shown in Table S3). The obtained products were analysed by agarose gel electrophoresis, purified using the Wizard® SV Gel and PCR Clean-Up System (Promega) or MinElute® PCR Purification Kit (Qiagen) and the technical replicates combined. PCR products from the same RNA sample were pooled and subjected to library preparation. ‘Confirmatory’ libraries were prepared using the TruSeq® DNA LT Sample Prep Kit with minor modifications, whereas ‘in depth’ libraries were prepared using the TruSeq® DNA Nano Library Kit (Illumina) according to the manufacturer’s instructions. Libraries were mixed with 50% PhiX Control Library (Illumina) and sequenced on the MiSeq System (Illumina), acquiring 151 bp read length. Of note, for the ‘confirmatory’ HeLa and Prostate datasets, only a single biological replicate for each sample was analysed, except for PrEC and LNCaP wild-type samples, which were performed in biological duplicates. For the ‘in depth’ HeLa dataset, all analyses were performed in biological triplicates.

##### Mapping of amplicon-bsRNA-seq reads and site calling

The target mRNA regions of all amplicons were combined into a single reference and the forward strand C-to-T converted. Reads from ‘confirmatory’ libraries were first trimmed using Trimmomatic [87] in palindromic mode with parameters (ILLUMINACLIP:illuminaClipping.fa:4:30:10:1:true LEADING:3 TRAILING:15 SLIDINGWINDOW:4:15 MINLEN:36). Sequencing reads were aligned using Bowtie2 within Bismark [88]. Reads from ‘in-depth’ sequencing were subjected to FastQC (v0.11.5); adapter removal and low-quality base trimming was performed using cutadapt (v1.18) with options (-q 20,20 -m 50 --trim-n -a AGATCGGAAGAGCACACGTCTGAACTCCAGTCA -A AGATCGGAAGAGCGTCGTGTAGGGAAAGAGTGT). Clean reads were aligned to the reference using the MeRanT tool in meRanTK [89] in both directions, as sequencing was not strand-specific, and the resulting bam files merged. Non-conversion level was determined for each C position within the amplicons.

#### Polysome bisulfite RNA sequencing data generation and analysis

##### Sucrose density gradient centrifugation and polysome profiling

For polysome profiling, HeLa cells were grown until ~ 70% confluent and then two technical replicate 150-mm-diameter plates were seeded with 6 × 10^6^ cells per biological replicate. Cells were again grown to ~ 70% confluency, and lysates prepared in the presence of cycloheximide (200 μl lysis buffer per plate), essentially as previously described [70]. The lysate protein concentrations were measured by *DC* protein assay (Bio-Rad) according to the manufacturer’s instructions and by absorption at 260 nm. Lysates were frozen and stored at − 80 °C until further processing. To recover the maximum amount of RNA possible, the entire lysate volume (~ 500 μl) was layered on top of a 10 ml 17.5–50% linear sucrose density gradient tube and separated by ultracentrifugation. Then, fractions were collected from the top of the gradient using a Brandel Gradient Fractionator (flow rate of 0.75 ml/min), collecting 24 fractions at 36-s intervals while measuring the absorption at 254 nm. The 10-μl subsamples were removed from each of the 24 fractions and combined by three to obtain a total of 8 protein fractions for downstream protein distribution analysis by Western blotting (as described above). All 24 collected fractions (0.5 ml) were spiked-in with 1 ng in vitro humanised *R-Luc* transcript to control for variations in RNA isolation efficacy. RNA was precipitated from each fraction with 1.5 ml ethanol in the presence of 5 μl glycogen (10 mg/ml) at − 80 °C overnight before proceeding to RNA extraction. RNA samples were combined by two to obtain 12 RNA fractions and subjected to a second ethanol precipitation step to remove residual sucrose. Yield and integrity of the isolated RNA was assessed using the RNA 6000 Nano Kit on the 2100 Bioanalyzer (Agilent Technologies). Three independent biological replicates were prepared in this way, with at least one cell passage between each. Key parameters for each replicate can be found in Table S1.

##### Reverse transcription and quantitative PCR (RT-qPCR)

DNA contamination was removed from RNA using the TURBO DNA-*free*™ Kit (Invitrogen) according to the manufacturer’s instructions. RNA was purified by phenol/chloroform extraction and precipitated. A subsample from each RNA fraction was reverse-transcribed using SuperScript™ III Reverse Transcriptase (Invitrogen) with oligo-dT primers, and qPCR amplifications were performed in technical triplicates using Fast SYBR® Green Master Mix (Applied Biosystems) in a 384-well format using the QuantStudio™ 12 K Flex system (Life Technologies). Primer sequences are listed in Table S3. The in vitro *R-Luc* spike-in transcript was used as control for variation in RNA isolation efficiency between each fraction and all results are represented relative to *R-Luc*.

##### Bisulfite conversion and bsRNA-seq

The remaining RNA was combined into four final bsRNA-seq fractions (Fig. 1), and 10 μg RNA was spiked with ERCC Spike-in Mix 2 (Ambion) according to the manufacturer’s instructions. RNA was then used for sequencing library construction using the TruSeq® Stranded Total RNA Library Kit (Illumina) according to the manufacturer’s instruction, with some modifications. Following rRNA depletion, RNA was suspended in nuclease-free water and subjected to bisulfite conversion as described previously (T Sibbritt, A Shafik, SJ Clark and T Preiss) [86]. Converted RNA was purified, and 1 μg used for continued library preparation with omission of the fragmentation step as the RNA undergoes fragmentation during the bisulfite treatment. No size selection was performed as the size of the bisulfite treated RNA fragments was between 50 and 200 nt with a peak size of approximately 150 nt (Fig. S1). The libraries were mixed equally and loaded onto the HiSeq 2500 System (Illumina) using a total of three lanes for sequencing. Sequencing was performed in fast mode acquiring 101-bp paired-end reads.

##### Preparation of reference sequences

The human reference genome hg38 and the GENCODE v28 annotation for each chromosome were downloaded from UCSC. The human ribosomal DNA complete repeating unit (U13369.1) was downloaded from NCBI and the human hg38 tRNA annotations were downloaded from GtRNAdb [90]. To assemble a pre-tRNA reference, 5′ and 3′ genomic flanking regions of length 100 nt with the corresponding tRNA reference were extracted from the genome with BEDTools. Intronic sequences were also included. ERCC spike-in reference sequences (SRM374) were obtained from www-s.nist.gov, and the *R-Luc* sequence is listed in Table S2. The reference genome sequence as well as ERCC and *R-Luc* spike-in sequences were combined into a single reference sequence, then converted as follows: C-to-T conversion of the forward strand and G-to-A conversion of the reverse strand followed by indexing using ‘meRanG mkbsidx’. tRNA and rRNA sequences were each treated as separate references and only C-to-T converted followed by indexing using ‘meRanT mkbsidx’ (Fig. S2).

##### Initial read mapping of bsRNA-seq reads

Raw reads were subjected to FastQC (v0.11.5). Low-quality bases and adaptor sequences were removed using Trimmomatic (v0.36, [87]) with options (ILLUMINCLIP:Adapter.fa:2:30:10:8:true LEADING:3 TRAILING:3 SLIDINGWINDOW:4:20 MINLEN:50). The processed reads with length greater than 50 nt were defined as clean reads. Forward and reverse reads were C-to-T and G-to-A converted, respectively, and mapped to the appropriate converted reference using the meRanGh tool (align bsRNA-seq reads to reference using HiSat2) in MeRanTK [89]. Only uniquely mapped reads were retained and replaced by the original unconverted reads. Mapping parameters for each library are shown in Table S4.

##### Background removal, non-conversion site calling, and site annotation

For transcriptome-wide candidate m^5^C site discovery, data from individual bsRNA-seq fraction libraries were pooled into their respective biological replicate (repB, repC, repE). To remove background non-conversion, reads containing more than three unconverted cytosines were removed from the bam files (‘3C’ filter; see also Table S4). Read counts at each cytosine position in the genome were obtained using the ‘mpileup’ function in samtools. Non-conversion sites were determined using a custom script with parameters ‘-minBQ 30 --overhang 6’. We observed a cytosine bias of uncertain origin at the 5′ and/or the 3′ end of the reads and thus opted to mask non-converted cytosines within the terminal 6 nt of each read to avoid overestimation of non-conversion. Candidate sites with a signal-to-noise ratio ≤ 0.9 (3C/raw; ‘S/*N* ≥ 0.9’) were further flagged and suppressed. To retain high-confidence non-conversion sites, the following criteria were applied: (1) total read coverage of ≥ 30 (‘≥ 30RC’), (2) non-converted C of ≥ 5 (‘5C’), (3) C + T coverage ≥ 80% (‘80CT’). An average non-conversion of ≥ 10% across the biological triplicates (‘10MM’) was also required. Candidate sites were annotated to the longest transcript according to the GENCODE v28 annotation (UCSC) using a custom script and mapped to six features: 5′UTR, CDS, 3′UTR, intronic, ncRNA_exonic and ncRNA_intronic. The RNA transcript type for each candidate site was extracted simultaneously. All candidate sites that could not be annotated were considered to be intergenic.

##### Read mapping and non-conversion site calling in tRNAs and rRNAs

For the tRNA analysis, reads were mapped to the pre-tRNA reference using the meRanT tool within meRanTK [89] with parameter (-k 10). Mapped reads containing more than three unconverted cytosines were removed from the bam files (3C filter) and only reads mapping to the predicted mature tRNA regions were retained (called ‘processed’ reads). tRNA sites were called as described for transcriptome-wide sites using only processed tRNA reads (e.g. sites shown in Fig. S4C,D). For rRNA analysis, reads were mapped to the ribosomal DNA complete repeat unit using meRanT tool within meRanTK. rRNA sites were called as described for transcriptome-wide site and using all reads mapped to the rRNA reference, excluding reads containing more than three unconverted cytosines, i.e. potentially including unprocessed precursors. All rRNA-related sites are reported in Fig. S4B and Table S5A, while only sites in mature rRNA regions are shown in Fig. S3C.

#### Candidate site characteristics and metagene analyses

##### Metagene distribution analysis of candidate m^5^C sites

Only exonic protein-coding candidate sites were used for distribution analysis along mRNAs. The relative position of each candidate site in the corresponding transcript feature (5′UTR, CDS or 3′UTR) was identified. For metagene density plots, each transcript feature was assigned a value corresponding to average feature length fraction out of the over-all transcript length. The background C control was generated using all C positions within the interrogated transcript segment of genes with candidate sites. Spatial enrichment analyses were conducted using RNAModR [78] with all unmodified C within the analysed region serving as background control. RNAModR was run using the GENCODE v20 annotation (UCSC) to build the transcriptome (DOI: 10.18129/B9.bioc.BSgenome.Hsapiens.UCSC.hg38).

##### Motif enrichment analysis

To acquire the sequence preference proximal to candidate sites, 21-nt sequences centred around each candidate site were extracted from the genome with BEDTools [91]. All C positions from genes with candidate sites were used as background control. Sequence logo plots were generated with ggseqlogo [77].

##### Secondary structure analysis

Secondary structure analysis surrounding the candidate mRNA sites was done as described previously [48]. Specifically, 25-nt sequences upstream and downstream of each candidate site were extracted from the genome with BEDTools [91] and folded with the RNAfold tool in the ViennaRNA Package 2.0 [76] using default parameters. The percentage of paired bases at each position was calculated from the folding results.

##### Gene ontology (GO) analysis

Gene Ontology (GO) analysis was performed using the *enrichGO* functionality within the clusterProfiler package [92]. Genes with FKPM ≥1 in the bsRNA-seq dataset were used as background control gene set. Resulting enriched GO terms were restricted to Bonferroni-corrected *p* values < 0.05.

##### Codon position bias of candidate sites

The codon position of each candidate site was calculated using various numpy functions in python from arrays of codon counts for each transcript. Arrays were obtained by counting all codons with a candidate site at the first, second or third position and comparison to all used codons. The enrichment of each codon was calculated using Fisher’s exact test.

##### Translation efficiency and mRNA stability analyses

For analysis of translation efficiency and mRNA half-life, publicly available HeLa datasets GSE49339 [33] and GSE102113 [79] were downloaded from NCBI. For analysis of translation efficiency during the somatic cell cycle (Asynchronous, S phase and M phase), the publicly available dataset GSE79664 [80] was also obtained. To quantify RNA or ribosome-protected fragment (RPF) abundance, annotations with more than 60 mapped reads were selected and normalised using the Trimmed Mean of M values (TMM) method implemented in the edgeR Bioconductor package [93]. Translation efficiency was calculated by dividing TMM normalised RPF values to that of RNA.

Cumulative density of translation efficiency and mRNA half-life were generated using genes containing candidate sites versus all expressed genes in HeLa cells.

##### Clustering of candidate sites across bsRNA-seq fractions

Only candidate sites in protein-coding genes were taken forward to clustering analyses. Candidate sites with any coverage in 9 out of 12 bsRNA-seq fraction samples and an average coverage ≥ 10 across the biological triplicates in each bsRNA-seq fraction were considered (F1234 clustering). As some candidate sites do not have enough coverage in bsRNA-seq fraction 1 (average coverage < 10 in F1), clustering was performed again considering only bsRNA-seq fractions 2–4 (F234; see Fig. S2). Average non-conversion level for each candidate site per bsRNA-seq fraction was used as input data. Clustering was performed using the Mfuzz soft clustering method [81] with the fuzzifier and cluster number parameters set to *m* = 2 and *c* = 9, respectively.

##### Logistic regression of non-conversion change between bsRNA-seq fractions

Sequential pairwise non-conversion level comparison between bsRNA-seq fractions F1 and F2, or F2 and F3, or F3 and F4 was carried out. Only sites with average coverage ≥ 10 in both bsRNA-seq fractions analysed were considered. Information from each fraction is specified (the average number of methylated Cs and average number of unmethylated Cs at a given site), and a logistic regression test was applied to compare the proportion of methylated Cs across two fractions using the methylKit R package [94]. Sites with a *q*-value < 0.05 and relative methylation difference ≥ 10% were defined as differentially methylated sites.

##### Statistical analysis

All bioinformatics-associated statistical analyses (unless stated otherwise) were performed in R. *p* < 0.05 is considered as statistically significant. Significance of average non-conversion changes were assessed by unpaired, two-tailed Students’ *t* test. Significance for candidate site distributions across mRNA regions within sampling-dependent pools (Figs. 5, S10) were assessed using binomial testing.

All significance levels are as follows: ns - not significant, *p < 0.05, **p < 0.01, ***p < 0.001, ****p < 0.0001.

## Supplementary information

Additional file 1: **Table S1.** HeLa cell lysate parameters. Measurements of HeLa cell lysate biological triplicates. For each biological replicate three technical replicate 150 mm diameter plates were seeded with six million (M) cells and grown for 24 h before harvest. Two plates were used for cell lysate preparation and the protein concentration measured by protein assay and absorption at 260 nm. The third plate was used to count final cell number.

Additional file 2: **Table S2.** Sequences of the Renilla Luciferase in vitro spike-in transcripts.

Additional file 3: **Figure S1.** Quality controls for polysome profiling and bsRNA-seq sample preparation. Related to Fig. 1. A: Distribution of tRNA and rRNA across gradients. Equal proportions of total RNA from each RNA fraction was analysed by microfluidic electrophoresis (Bioanalyzer RNA 6000 Nano Chip; equal proportions of recovered RNA were loaded). Pseudo-gel images for each of the three biological replicates are shown. B: Distribution of additional representative mRNAs across gradients. mRNA levels in each RNA fraction were determined by RT-qPCR. Results for three mRNAs of different coding region length are shown: *RPL13a* (ribosomal protein L13a), *MAP 2 K2* (mitogen-activated protein kinase kinase 2) and *NDUFB7* (NADH: ubiquinone oxidoreductase subunit B7). mRNA levels per fraction were normalised to the level of a spike-in control, rescaled as percentage of total signal across all fractions, and are shown as mean ± standard deviation across the three biological replicates. A representative absorbance trace (254 nm) is shown at the top for reference. C: RNA quality of bsRNA-seq fractions prior to bisulfite treatment. RNA from each bsRNA-seq fraction was analysed by microfluidic electrophoresis (Bioanalyzer RNA 6000 Nano Chip; an equal amount of RNA was loaded per well). Pseudo-gel images for each of the three biological replicates are shown. D: Microfluidic electrophograms for biological replicate E tracing the RNA quality at each step from input to the final library (from left to right). Data shown are exemplary for all biological replicates.

Additional file 4: **Table S3.** Primers used in this study.

Additional file 5: **Figure S2.** bsRNA-seq mapping and data analysis. Related to Figs. 2 and 5. Workflow from bsRNA-seq read processing and mapping, m^5^C candidates site selection to clustering by non-conversion level across polysome gradients. For the definitive site selection, steps in the workflow were performed sequentially. Selection criteria for high confidence candidate sites and alternate groupings of bsRNA-seq libraries for different purposes are indicated. Note, four bsRNA-seq fraction libraries representing distinct translation states were sequenced per biological replicate, creating a total of twelve libraries termed LibB1–4, LibC1–4 and LibE1–4. For global m^5^C candidate site calling, Libs 1–4 were combined into one composite library for each biological replicate, creating cLibB, C and E. These composite libraries approximate a total transcriptome-wide survey for each biological replicate. For clustering analyses, libraries from corresponding bsRNA-seq fractions (i.e. LibB1, LibC1 and LibE1 and so forth) formed biological replicates of each other.

Additional file 6: **Table S4.** Mapping statistics of all 12 libraries and the combined replicates. Statistics are given for mapping to the genome, tRNA and rRNA sequences and ERCC and R-Luc spike-in.

Additional file 7: **Figure S3.** Effects of the 3C and S/N90 filters on specificity and sensitivity of m^5^C candidate site detection. Related to Fig. 2. In each panel, plots are arranged vertically by RNA under investigation, and horizontally by the extent of sequential filtering (initial read mapping—after removing reads with > 3 non-converted cytosines ‘3C filter’—after suppressing sites below the chosen signal-to-noise threshold ‘3C & S/N90 filter’ [less than 90% of reads passing the 3C filter]). Dual y-axis plots show either cytosine conversion (A,C) or non-conversion (B) (left y-axis, blue bars) and read coverage (right y-axis, red line) against cytosine position in the respective reference sequence (x-axis). Data is shown as mean across the three biological replicates with error bars indicating ± standard deviation. Candidate sites disqualified by the S/N90 filter are identified by orange bars. The effects of the filters were evaluated using selected spike-in control (A), rRNA (B) and tRNA (C) sequences. A: Panel of spike-in controls, *R-Luc* RNA and two arbitrarily selected ERCC transcripts. B: Mature ribosomal RNA species. Note that cytosine non-conversion is plotted for improved visualisation. The fourth to sixth panels show zoomed-in plots of fully filtered 18S and 28S rRNA data. Residues of zoomed regions are indicated on the top and correspond to numbering in full-scale plots. Green arrows and position labelling indicate the two known m^5^C sites in 28S rRNA [96]. C: Selected tRNA examples. tRNA^Asp^ (GUC), tRNA^Glu^ (UUC) and tRNA^Gly^ (GCC) were chosen to represent different m^5^C positions within tRNAs and to illustrate the adverse effect of the chosen filters on tRNAs with > 3 modified cytosines. Cytosine numbering is according to the tRNA consensus structural positions.

Additional file 8: **Table S5.** A: Candidate sites detected in ribosome RNA repeat unit. B: Candidate sites detected transcriptome-wide. Candidate sites in red have been analysed by amplicon bsRNA-seq. C: Candidate sites detected in mature tRNA transcripts.

Additional file 9: **Figure S4.** m^5^C candidate site call reproducibility across biological replicates and effects of non-conversion ‘noise suppression’. Related to Fig. 2. A: Pair-wise scatter plot comparisons of transcriptome-wide candidate sites called in composite libraries of each biological replicate. Sites shown passed the 80CT, 30RC and 5C filter in their respective composite library (a non-conversion cut-off was not applied). Further to that, only sites with coverage in all three replicates were used. The adjusted R-squared value following linear regression is shown. B: Effect of the 3C and S/N90 filters on candidate site calling in different RNA types. The number of candidate sites that passed the 80CT, 30RC, 5C filter in their respective composite library and fulfilled the 10MM criterion are listed. C: Position of candidate sites in the tRNA cloverleaf consensus structure. Each circle indicates a nucleotide position within the tRNA cloverleaf structure, with blue filled circles indicating position at which candidate sites were identified. Iso-acceptors found to carry the candidate site are identified by the single letter amino acid code. D: Genetic code table highlighting tRNA iso-decoders with candidate sites in blue. C-D: Of note, we detected the NSUN2-dependent sites at the edge of the variable loop at position C48–50 in a variety of tRNA iso-decoders, as well as at position C34 of intron-containing tRNA^Leu^ (CAA). We further identified the TRDMT1-dependent modification of C38 in tRNA^Asp^ (GUC). Interestingly, we also detected several candidate sites at structural position C72. The established NSUN6-dependent sites in tRNA^Thr^ (UGU) and tRNA^Cys^ (GCA) iso-decoders did not receive read coverage. Instead, we saw clear non-conversion at C72 in tRNA^Ile^ (UAU), tRNA^Lys^ (CUU) and tRNA^Ser^ (ACU); these might be novel NSUN6 substrates. C70 in tRNA^Gly^ (CCC) is indicated in the figure, however, detection of this site is heavily driven by the terminal base of reads in one direction, thus likely suspect.

Additional file 10: **Figure S5.** siRNA knockdown efficiency controls. Related to Fig. 3. HeLa cells, or the prostate cell lines LNCaP or PrEC, were transiently transfected with siRNAs targeting the m^5^C:RNA methyltransferases NSUN2 or TRDMT1, the m^5^C:DNA methyltransferase DNMT1 or a non-targeting control (NTC) as indicated in the panels. Across panels, results are arranged with HeLa ‘confirmatory’ data on top (*N* = 1), HeLa ‘in depth’ data in the middle (*N* = 3), and prostate cell line ‘confirmatory’ data at the bottom (N = 1). A: Western blots for NSUN2, TRDMT1, DNMT1 and the internal controls alpha-tubulin or GAPDH are indicated on the left. siRNA knockdown condition is shown below. One replicate for the HeLa ‘in depth’ data is shown; similar results were obtained for the other replicates. B: mRNA levels are shown relative to those in the NTC control. HeLa RT-qPCR data were normalised to the internal control genes *HPRT* (top) or *GAPDH* (middle), RT-qPCR data from the prostate cell lines were normalised to the geometric mean of the internal control genes *MRPL9*, *H2AFV* and *TCF25*. ‘Confirmatory’ data (top and bottom) are shown as averages of three technical replicates. ‘In depth’ data (middle) are shown as averages of three biological replicates. Error bars indicate standard error of the mean. C-D: Amplicon-bsRNA-seq results for the *R-Luc* spike-in negative controls (C) and selected tRNAs as positive controls (D). Grids are organised by knockdown sample in rows and cytosine position along the analysed transcript section in columns (for tRNAs structural positions are given for the first interrogated cytosine as well as the candidate m^5^C sites [in red]). A white-to-red colour scale is used to tint each square by the degree of cytosine non-conversion. Note, C38 in tRNA^Gly^ (GCC) is a known target of TRDMT1, whereas C48–50 in tRNA^Gly^ (GCC) and C48 in tRNA^Thr^ (UGU) are mediated by NSUN2. Student’s *t*-test results of non-conversion change for tRNA^Gly^ (GCC) in the ‘confirmatory’ HeLa data is indicated next to the grid with asterisks and diamonds showing significance to the NSUN2 or TRDMT1 knockdown sample, respectively (key in top panel).

Additional file 11: **Figure S6.** Additional validation and NSUN2-dependence of candidate m^5^C sites in mRNA and ncRNA. Related to Fig. 3. Amplicon-bsRNA-seq was performed with total RNA isolated from HeLa cells, or the prostate cell lines LNCaP or PrEC, after siRNA-mediated m^5^C:RNA methyltransferase knockdown targeting NSUN2 or TRDMT1 along with control siRNAs (targeting m^5^C:DNA methyltransferase DNMT1 or a non-targeting control [NTC]; see Fig. S5 for knockdown efficiency controls). Read coverage per amplicon was from 3500 to 67,500 (see Table S6). Grids are organised by knockdown sample in rows and cytosine position along the analysed transcript section in columns (genomic coordinates are given for the first and last interrogated cytosine position [in black], as well as the candidate m^5^C site [in red]). A white-to-red colour scale is used to tint each square by the degree of cytosine non-conversion. The longest mRNA isoform (based on Ensembl) is shown with the candidate m^5^C site position indicated by a red circle. The sequence context (non-converted cytosine indicated in red) is given. The enzyme identified to be responsible for cytosine methylation is indicated above the candidate sites: N – NSUN2; N? – unresolved but likely NSUN2. A: ‘Confirmatory’ HeLa data (*N* = 1) shown for mRNA sites in *NAPRT* (nicotinate phosphoribosyltransferase), *CINP* (cyclin dependent kinase 2 interacting protein), *SCO1* (SCO cytochrome C oxidase assembly protein 1) and *MCFD2* (multiple coagulation factor deficiency 2) (top and middle row). ‘In depth’ data (*N* = 2–3) is shown as the average of at least two biological replicates for mRNA sites in *CCT5* (chaperonin containing TCP1 Subunit 5) and *NSUN2* (bottom row). These two sites are likely controlled by NSUN2, although the non-conversion change is not significantly (ns) different (Student’s *t*-test) in comparison to the NSUN2 KD sample (non-conversion averages are indicated to the right; see also Table S6). B: ‘Confirmatory’ prostate data (N = 1) shown for mRNA sites in *SZRD1* (SUZ RNA binding domain containing 1), *RTN3* (reticulon 3), *PWP2* (PWP2 small subunit processome component) and *SRRT* (serrate) (top and middle row) and ncRNA sites in *SCARNA2* (small Cajal body-specific RNA 2) and *SNORD62B* (small nucleolar RNA, C/D box 62B) (bottom row).

Additional file 12: **Table S6.** Description of candidate sites analysed by amplicon bsRNA-seq. Each site is identified by genomic coordinates. Average non-conversion (MM) and average coverage (MCov) is given for the dataset indicated at the top. The Methyltransfrase identified to be responsible for methylation along with the sequence context of the candidate site are also indicated.

Additional file 13: **Figure S7.** Sequence context and NSUN2-dependence of candidate m^5^C sites. Related to Fig. 4. A: Gene Ontology (GO) term enrichment of candidate sites. Analysis was performed using the enrichGO function in ClusterProfiler using transcriptome-wide candidate sites (*n* = 846), with all genes detected at FKPM≥1 in the bsRNA-seq used as background. Bonferroni correction was applied and terms with *p* < 0.05 deemed enriched. The ten most enriched terms for Cellular Component (left) and Biological Process (right) are shown (for full list see Table S7). No enrichment was obtained for Molecular Function GO terms. B: Codon position enrichment analysis of candidate sites within the CDS of protein-coding genes. All three codon positions were analysed and are indicated at the top. Codons preferentially containing candidate sites are indicated in red, with significance following Fisher’s exact test indicated: ns - not significant. C: Sequence context of candidate sites (top) in comparison to all cytosines in the same transcripts (bottom). Logos were generated for candidate sites present within the four RNA transcript regions (5′UTR: m^5^C, *N* = 91; Null = 150,943. CDS: m^5^C, *N* = 410; Null = 1,139,649. 3′UTR: m^5^C, *N* = 201; Null = 589,922. Intronic: m^5^C, *N* = 152; Null = 26,326,026). All sites from ‘protein-coding’ (*N* = 846) and ‘NMD’ RNA biotypes (N = 8) were included. D: Spatial enrichment analyses of candidate sites within mRNAs. Site are placed into bins as indicated on the x-axis. Site distribution across bins is compared to matching randomised cytosine sampling (Null) and the log10 Odds-ratio (OR) is plotted as a red line with the 95% confidence interval (CI) shaded. Significance of enrichment is plotted as the log10 *p*-value by blue bars: ns - not significant. Analyses were anchored at either the transcription start (left) or the transcription end (right) and performed using RNAModR with a bin width of 100 nt and a window of 400 nt for the 5′UTR or 1000 nt for the 3′UTR region.

Additional file 14: **Table S7.** GO annotation of candidate transcriptome-wide m^5^C sites identified in polysome bsRNA-seq.

Additional file 15: **Figure S8.** Correlation of mRNA translation and stability with m^5^C site content. Related to Fig. 4. A: Cumulative density distribution of translation efficiency for candidate site-containing and all remaining mRNAs. Translation efficiency values are based on HeLa cell ribosome profiling data from [X Wang, ZK Lu, A Gomez, GC Hon, YN Yue, DL Han, Y Fu, M Parisien, Q Dai, GF Jia, et al. [33]; first plot], [[79]; second plot] or [[80]; cell cycle plots]. (GSE49339: m^5^C mRNA, *N* = 666; remaining mRNA, *N* = 12,185. GSE102113: m^5^C mRNA, *N* = 667; remaining mRNA, *N* = 11,460. GSE79664: m^5^C mRNA, *N* = 669; remaining mRNA, N = 11,717). B: Cumulative density distribution of mRNA half-life for candidate site-containing and all remaining mRNAs. HeLa cell mRNA half-life data was taken from [[33]; first plot)] or [[79]; second plot]. (GSE49339: m^5^C mRNA, *N* = 649; remaining mRNA, *N* = 10,569. GSE102113: m^5^C mRNA, *N* = 612; remaining, N = 11,175).

Additional file 16: **Table S8.** A: Non-conversion level of high confidence candidate site across the four gradient fractions. Sites marked in red were assessed by amplicon bsRNA-seq. B: Clustering of protein-coding candidate sites across all four gradient fractions - F1234. Sites marked in red were assessed by amplicon bsRNA-seq. C: Clustering of protein-coding candidate sites across three gradient fractions - F234. Sites marked in red were assessed by amplicon bsRNA-seq. D: Differential methylation analysis by linear regression test across the gradient fractions. All high confidence candidate sites with sufficient coverage are included in this analysis. Sites with FDR < 0.05 are highlighted in red, sites with ≥10% relative negative or positive change are highlighted in green and yellow, respectively.

Additional file 17: **Figure S9.** Clustering of candidate m^5^C site non-conversion level patterns across the polysome profile. Related to Fig. 5. bsRNA-seq libraries were grouped as biological triplicates per fraction (e.g. LibB 1, LibC1 and LibE1 each report on sites detected in bsRNA-seq fraction 1), allowing the calculation of average non-conversion levels per individual site and per fraction. Non-conversion levels per individual site across the polysome profile were partitioned into nine soft clusters (C1–9 arranged here by similarity in trend with polysome profile) using Mfuzz. 254 candidate m^5^C sites were included based on having coverage in at least 9 out of 12 bsRNA-seq fraction samples and ≥ 10 average coverage in each of the four bsRNA-seq fractions. Degree of cluster ‘membership’ is indicated by the colour scale depicted to the right of panel A. Candidate sites with high membership (blue) have the best match to the respective cluster’s overall pattern. The legend to the right gives a colour/significance key applicable to all panels. Insets are: pie charts showing distribution of cluster members across different mRNA regions; boxplots showing site non-conversion distribution of cluster members across the polysome profile. Asterisks indicate significance *p*-value from unpaired, two-tailed Student’s *t*-test comparing the means of adjacent fractions. For Fig. 5, clusters C9,2,7,1,4,6 were combined into the negative trend category, clusters C3,5 into the neutral category, and cluster C8 formed the positive category. (PDF 944 kb)

Additional file 18: **Figure S10.** Relationship between non-conversion level and mRNA translation state for candidate m^5^C sites with insufficient coverage in bsRNA-seq fraction 1. Related to Fig. 5. bsRNA-seq libraries were grouped as biological triplicates per fraction (e.g. LibB 1, LibC1 and LibE1 each report on sites detected in bsRNA-seq fraction 1), allowing the calculation of average non-conversion levels per individual site and per fraction. A: Non-conversion levels per individual site across the polysome profile were partitioned into nine soft clusters (C1–9 arranged here by similarity in trend with polysome profile) using Mfuzz. 315 candidate m^5^C sites were included based on having coverage in at least 9 out of 12 bsRNA-seq fraction samples and ≥ 10 average coverage in bsRNA-seq fractions 2–4 but failing this criterion for fraction 1. Degree of cluster ‘membership’ is indicated by the colour scale depicted to the right of panel A. Candidate sites with high membership (blue) have the best match to the respective cluster’s overall pattern. The legend to the right of panel A gives a colour/significance key applicable to all panels. Insets are: pie charts showing distribution of cluster members across different mRNA regions; boxplots showing site non-conversion distribution of cluster members across the polysome profile. Asterisks indicate significance p-value from unpaired, two-tailed Student’s *t*-test comparing the means of adjacent fractions. B: Mfuzz clusters from panel A were grouped into three translation state trend categories by visual inspection, showing a negative (clusters C3,4,6,9; *N* = 133), neutral (clusters C2,8,1; *N* = 75) or positive trend (clusters C5,7; *N* = 107) with polysome association. Top panels: line graphs displaying individual site average non-conversion levels across fractions. Middle panels: boxplots showing distribution of site non-conversion levels in each fraction. Asterisks indicate significance p-value from unpaired, two-tailed Student’s *t*-test comparing the means of adjacent fractions. Bottom panels: boxplots showing distribution of site coverage in each fraction. C: Stacked bar charts showing distribution of sites in the different translation state trend categories across mRNA regions (top) and distribution of sites in different mRNA regions across translation state trend categories (bottom). Asterisks indicate significance *p*-values following binomial test against the distribution of all sites. The legend to the right of panel A gives a colour/significance key applicable to all panels.

Additional file 19: **Figure S11.** Identification of individual sites with significant correlation of cytosine non-conversion with polysome co-sedimentation. Related to Fig. 6. A: Transcriptome-wide candidate m^5^C sites were used as input for logistic regression analysis (methylKit; [94]. For each pairwise comparison of adjacent polysome profile fractions a minimum of ≥10 average read coverage across each bsRNA-seq fraction was required. Plots show the -log10 q-value (FDR) of the non-conversion change, against the relative non-conversion difference for each pairwise comparison, with the number of sites that qualified indicated above (993 of 1034 sites were involved in at least one such comparison). Each dot represents a single site, sites that score as significant (q-value < 0.05) and showing a relative non-conversion change ≥10% are coloured in red (negative correlation) and green (positive correlation), respectively. B: Characteristics of sites included in the F1234 clustering (Figs. 5 and S9) that showed any significant trend as analysed in panel A. C-D: Examples of individual sites in mRNA showing significant correlation of cytosine non-conversion with translation state. Dual axis charts show cytosine non-conversion (bars) and coverage (red lines) for a given site across the polysome gradient. Data is shown as means across biological triplicates, with error bars indicating standard deviation. Asterisks indicate significance p-values after logistic regression testing (see key next to panel D). The gene name for each candidate site and its position within the mRNA is given. C: Individual examples with significant negative non-conversion change in at least two fraction steps. The cluster number from the F1234 clustering is indicated in brackets. Candidate sites shown here are from the following mRNAs: *PSMD3* (proteasome 26S subunit); *CSNK1A1* (casein kinase 1 alpha 1); *H2AFY* (H2A histone family member Y); *NELFCD* (negative elongation factor complex member C/D); *ADRM1* (adhesion regulating molecule 1); *APRT* (adenine phosphoribosyltransferase); *SMARCC* (SWI/SNF related, matrix associated, actin dependent regulator of chromatin subfamily C member 1); *AKR7A2* (aldo-keto reductase family 7 member A2); *SLC16A3* (solute carrier family 16 member 3); *APEH* (acylaminoacyl-peptide hydrolase); *DBNL* (drebrin like); *ETF1* (eukaryotic translation termination factor 1). D: Examples with significant positive non-conversion change in at least one fraction step. Candidate sites shown here are from the following mRNAs: *RPS29* (ribosomal protein 29); *RPSA* (ribosomal protein SA); *RPLP1* (ribosomal protein lateral stalk subunit P1); *RTEL1* (regulator of telomerase elongation helicase 1); *HYI* (hydroxypyruvate isomerase); *MCAM* (melanoma cell adhesion molecule).

## Data Availability

All source code used during this study is available at https://github.com/YangLab/bsRNA-seq-m5C. All raw and processed sequencing data generated in this study have been submitted to the NCBI Gene Expression Omnibus (GEO; https://www.ncbi.nlm.nih.gov/geo/) under accession number GSE140995 (https://www.ncbi.nlm.nih.gov/geo/query/acc.cgi?acc=GSE140995) [95].
