# Supplementary material for: Multiple links between 5-methylcytosine content of mRNA and translation
Source: BMC Biol. 2020 Apr 15;18:40. doi: 10.1186/s12915-020-00769-5 (PMC7158060; doi:10.1186/s12915-020-00769-5)
Supplement: Supplementary file 1 — Table S1. HeLa cell lysate parameters. Measurements of HeLa cell lysate biological triplicates. For each biological replicate three technical replicate 150 mm diameter plates were seeded with six million (M) cells and grown for 24 h before harvest. Two plates were used for cell lysate preparation and the protein concentration measured by protein assay and absorption at 260 nm. The third plate was used to count final cell number. [file 12915_2020_769_MOESM1_ESM.docx]

**Supplementary Table S1: HeLa cell lysate parameters.** Measurements of biological HeLa cell lysate triplicates. For each biological replicate three technical replicate 150mm diameter plates were seeded with six million (M) cells and grown for 24h before harvest. Two plates were used for cell lysate preparation and the protein concentration measure by protein assay and absorption at 260nm. The third plate was used to count final cell number.

|  | **Replicate B** | **Replicate C** | **Replicate E** |
| --- | --- | --- | --- |
| **Cell number** | ~23M | ~21M | ~24M |
| **Protein concentration** | 7.98 mg/ml | 7.79 mg/ml | 7.74 mg/ml |
| **Absorption at A260** | 17.45 U | 14.98 U | 13.07 U |
