# Supplementary material for: Multiple links between 5-methylcytosine content of mRNA and translation
Source: BMC Biol. 2020 Apr 15;18:40. doi: 10.1186/s12915-020-00769-5 (PMC7158060; doi:10.1186/s12915-020-00769-5)
Supplement: Supplementary file 2 — Table S2. Sequences of the Renilla Luciferase in vitro spike-in transcripts. [file 12915_2020_769_MOESM2_ESM.docx]

**Supplementary Table S2:** **Sequences of the Renilla Luciferase *in vitro* spike-in transcripts.**

**A:** Humanised R-Luc *in vitro* transcript sequence:

TCCAAGGTGTACGACCCCGAGCAACGCAAACGCATGATCACTGGGCCTCAGTGGTGGGCTCGCTGCAAGCAAATGAACGTGCTGGACTCCTTCATCAACTACTATGATTCCGAGAAGCACGCCGAGAACGCCGTGATTTTTCTGCATGGTAACGCTGCCTCCAGCTACCTGTGGAGGCACGTCGTGCCTCACATCGAGCCCGTGGCTAGATGCATCATCCCTGATCTGATCGGAATGGGTAAGTCCGGCAAGAGCGGGAATGGCTCATATCGCCTCCTGGATCACTACAAGTACCTCACCGCTTGGTTCGAGCTGCTGAACCTTCCAAAGAAAATCATCTTTGTGGGCCACGACTGGGGGGCTTGTCTGGCCTTTCACTACTCCTACGAGCACCAAGACAAGATCAAGGCCATCGTCCATGCTGAGAGTGTCGTGGACGTGATCGAGTCCTGGGACGAGTGGCCTGACATCGAGGAGGATATCGCCCTGATCAAGAGCGAAGAGGGCGAGAAAATGGTGCTTGAGAATAACTTCTTCGTCGAGACCATGTCCCAAGCAAGATCATGCGGAAACTGGAGCCTGAGGAGTTCGCTGCCTACCTGGAGCCATTCAAGGAGAAGGGCGAGGTTAGACGGCCTACCCTCTCCTGGCCTCGCGAGATCCCTCTCGTTAAGGGAGGCAAGCCCGACGTCGTCCAGATTGTCCGCAACTACAACGCCTACCTTCGGGCCAGCGACGATCTGCCTAAGATGTTCATCGAGTCCGACCCTGGGTTCTTTTCCAACGCTATTGTCGAGGGAGCTAAGAAGTTCCCTAACACCGAGTTCGTGAAGGTGAAGGGCCTCCACTTCAGCCAGGAGGACGCTCCAGATGAAATGGGTAAGTACATCAAGAGCTTCGTGGAGCGCGTGCTGAAGAACGAGCAGTAACGAGCAGTAA

**B:** Non-humanised R-Luc *in vitro* transcript sequence:

ATGATCCAGAACAAAGGAAACGGATGATAACTGGTCCGCAGTGGTGGGCCAGATGTAAACAAATGAATGTTCTTGATTCATTTATTAATTATTATGATTCAGAAAAACATGCAGAAAATGCTGTTATTTTTTTACATGGTAACGCGGCCTCTTCTTATTTATGGCGACATGTTGTGCCACATATTGAGCCAGTAGCGCGGTGTATTATACCAGACCTTATTGGTATGGGCAAATCAGGCAAATCTGGTAATGGTTCTTATAGGTTACTTGATCATTACAAATATCTTACTGCATGGTTTGAACTTCTTAATTTACCAAAGAAGATCATTTTTGTCGGCCATGATTGGGGTGCTTGTTTGGCATTTCATTATAGCTATGAGCATCAAGATAAGATCAAAGCAATAGTTCACGCTGAAAGTGTAGTAGATGTGATTGAATCATGGGATGAATGGCCTGATATTGAAGAAGATATTGCGTTGATCAAATCTGAAGAAGGAGAAAAAATGGTTTTGGAGAATAACTTCTTCGTGGAAACCATGTTGCCATCAAAAATCATGAGAAAGTTAGAACCAGAAGAATTTGCAGCATATCTTGAACCATTCAAAGAGAAAGGTGAAGTTCGTCGTCCAACATTATCATGGCCTCGTGAAATCCCGTTAGTAAAAGGTGGTAAACCTGACGTTGTACAAATTGTTAGGAATTATAATGCTTATCTACGTGCAAGTGATGATTTACCAAAAATGTTTATTGAATCGGACCCAGGATTCTTTTCCAATGCTATTGTTGAAGGTGCCAAGAAGTTTCCTAATACTGAATTTGTCAAAGTAAAAGGTCTTCATTTTTCGCAAGAAGATGCACCTGATGAAATGGGAAAATATATCAAATCGTTCGTTGAGCGAGTTCTCAAAAATGAACAATAATTCTAGAGCGGCCGCTTCGAGCAGACATGATAAGATACATTGATGAGTTTGGACAAACCACAACTAGAATGCAGTGAAAAAAATGCTTTATTTGTGAAATTTGTGATGCTATTGCTTTATTTGTAACCATTATAAGCTGCAATAAACAAGTTAACAACAACAATTGCATTCATTTTATGTTTCAGGTTCAGGGGGAGGTGTGGGAGGTTTTTTAAAGCAAGTAAAACCTCTACAAATGTGGTAAAATCGATAAGGATCCAAAAAAAAAAAAAAAAAAAAAAA
