# Supplementary figures and images for: Multiple links between 5-methylcytosine content of mRNA and translation
Source: BMC Biol. 2020 Apr 15;18:40. doi: 10.1186/s12915-020-00769-5 (PMC7158060; doi:10.1186/s12915-020-00769-5)

**Figure S1:**

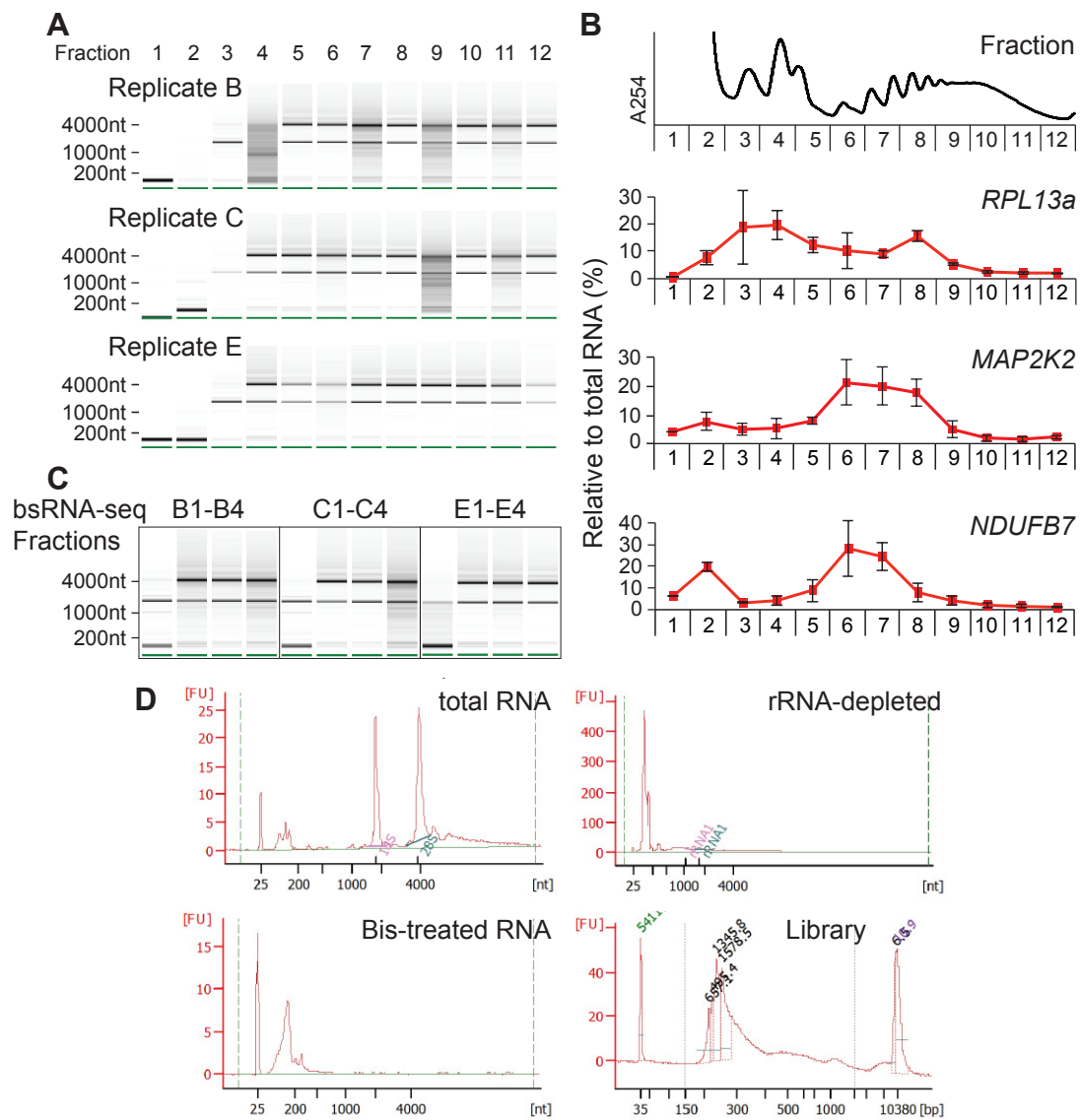

Supplement: Supplementary file 3 — Figure S1. Quality controls for polysome profiling and bsRNA-seq sample preparation. Related to Fig. 1. A: Distribution of tRNA and rRNA across gradients. Equal proportions of total RNA from each RNA fraction was analysed by microfluidic electrophoresis (Bioanalyzer RNA 6000 Nano Chip; equal proportions of recovered RNA were loaded). Pseudo-gel images for each of the three biological replicates are shown. B: Distribution of additional representative mRNAs across gradients. mRNA levels in each RNA fraction were determined by RT-qPCR. Results for three mRNAs of different coding region length are shown: RPL13a (ribosomal protein L13a), MAP 2 K2 (mitogen-activated protein kinase kinase 2) and NDUFB7 (NADH: ubiquinone oxidoreductase subunit B7). mRNA levels per fraction were normalised to the level of a spike-in control, rescaled as percentage of total signal across all fractions, and are shown as mean ± standard deviation across the three biological replicates. A representative absorbance trace (254 nm) is shown at the top for reference. C: RNA quality of bsRNA-seq fractions prior to bisulfite treatment. RNA from each bsRNA-seq fraction was analysed by microfluidic electrophoresis (Bioanalyzer RNA 6000 Nano Chip; an equal amount of RNA was loaded per well). Pseudo-gel images for each of the three biological replicates are shown. D: Microfluidic electrophograms for biological replicate E tracing the RNA quality at each step from input to the final library (from left to right). Data shown are exemplary for all biological replicates. [file 12915_2020_769_MOESM3_ESM.pdf]

**Figure S2:**

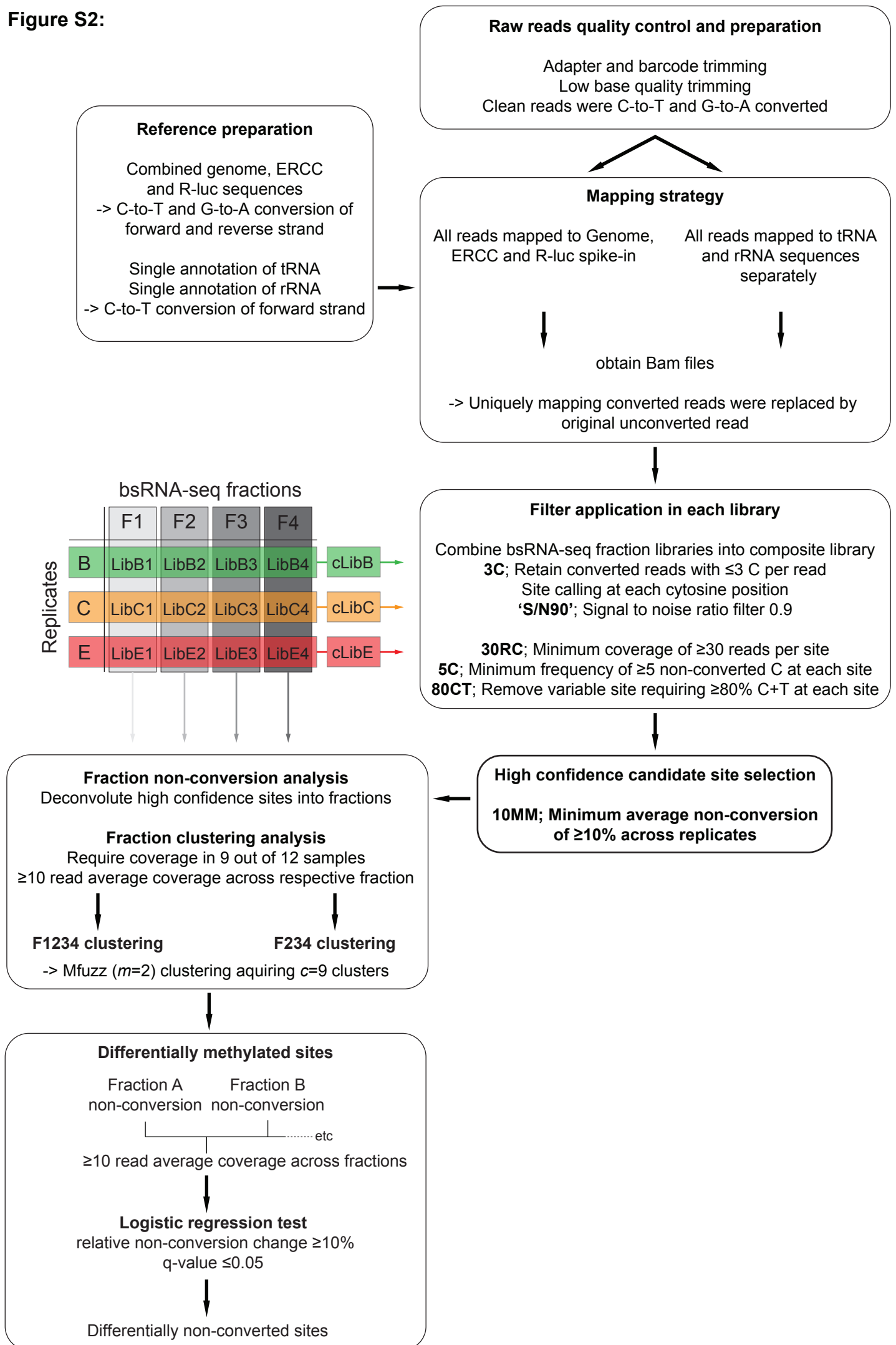

Supplement: Supplementary file 5 — Figure S2. bsRNA-seq mapping and data analysis. Related to Figs. 2 and 5. Workflow from bsRNA-seq read processing and mapping, m5C candidates site selection to clustering by non-conversion level across polysome gradients. For the definitive site selection, steps in the workflow were performed sequentially. Selection criteria for high confidence candidate sites and alternate groupings of bsRNA-seq libraries for different purposes are indicated. Note, four bsRNA-seq fraction libraries representing distinct translation states were sequenced per biological replicate, creating a total of twelve libraries termed LibB1–4, LibC1–4 and LibE1–4. For global m5C candidate site calling, Libs 1–4 were combined into one composite library for each biological replicate, creating cLibB, C and E. These composite libraries approximate a total transcriptome-wide survey for each biological replicate. For clustering analyses, libraries from corresponding bsRNA-seq fractions (i.e. LibB1, LibC1 and LibE1 and so forth) formed biological replicates of each other. [file 12915_2020_769_MOESM5_ESM.pdf]

Figure S3A:

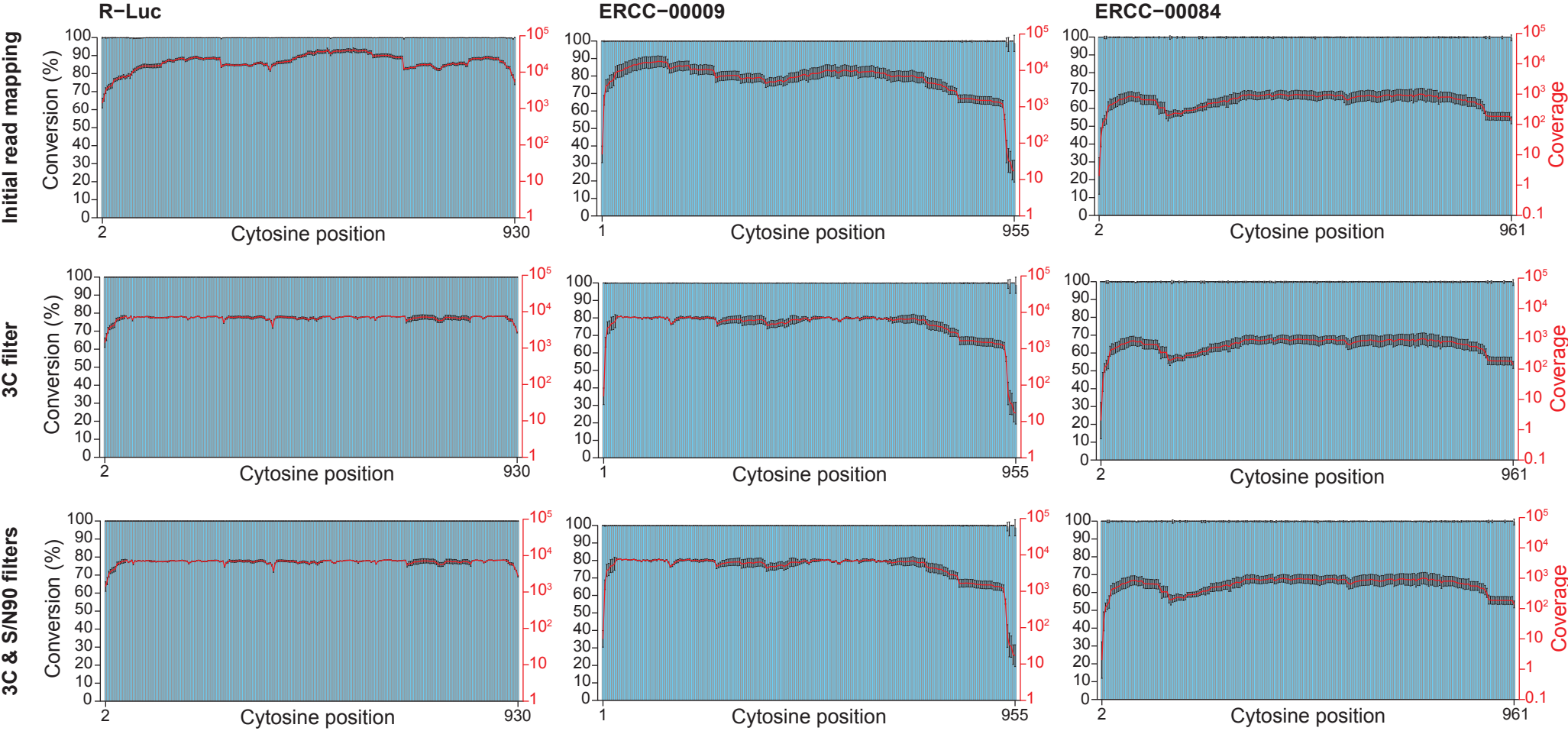

**Figure S3B:**

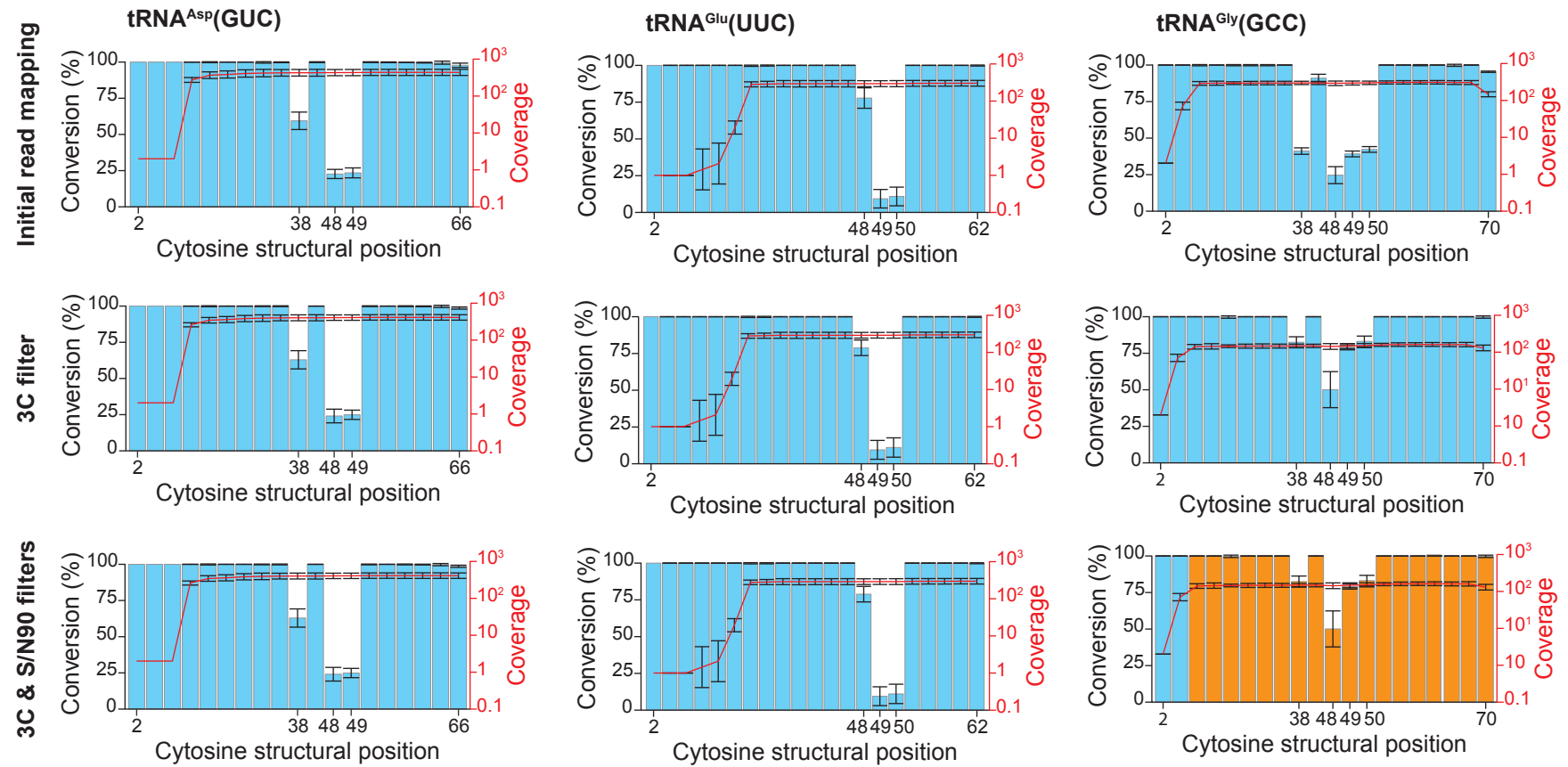

Figure S3C:

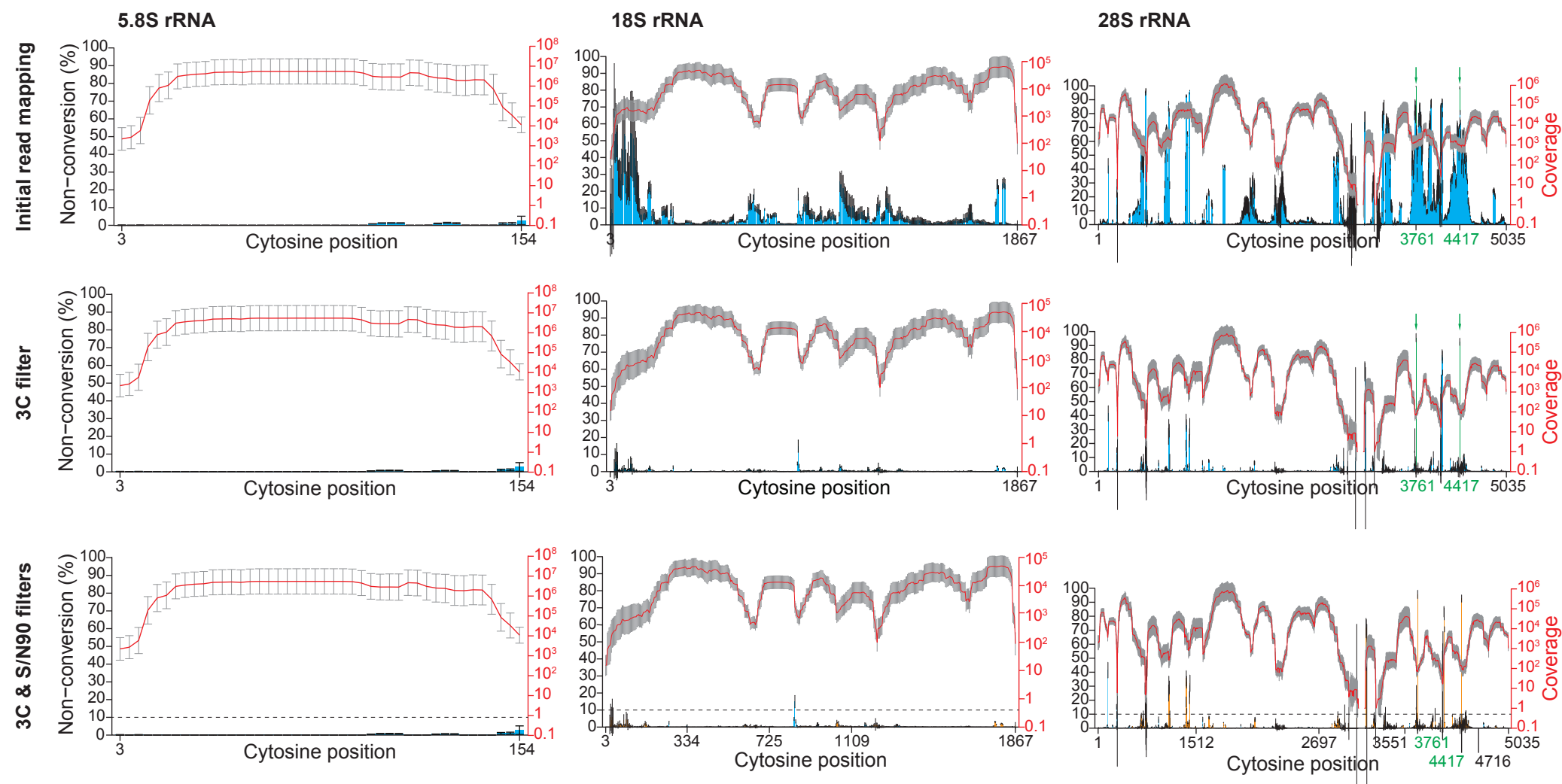

Figure S3C continued:

18S and 28S rRNA zoomed plots

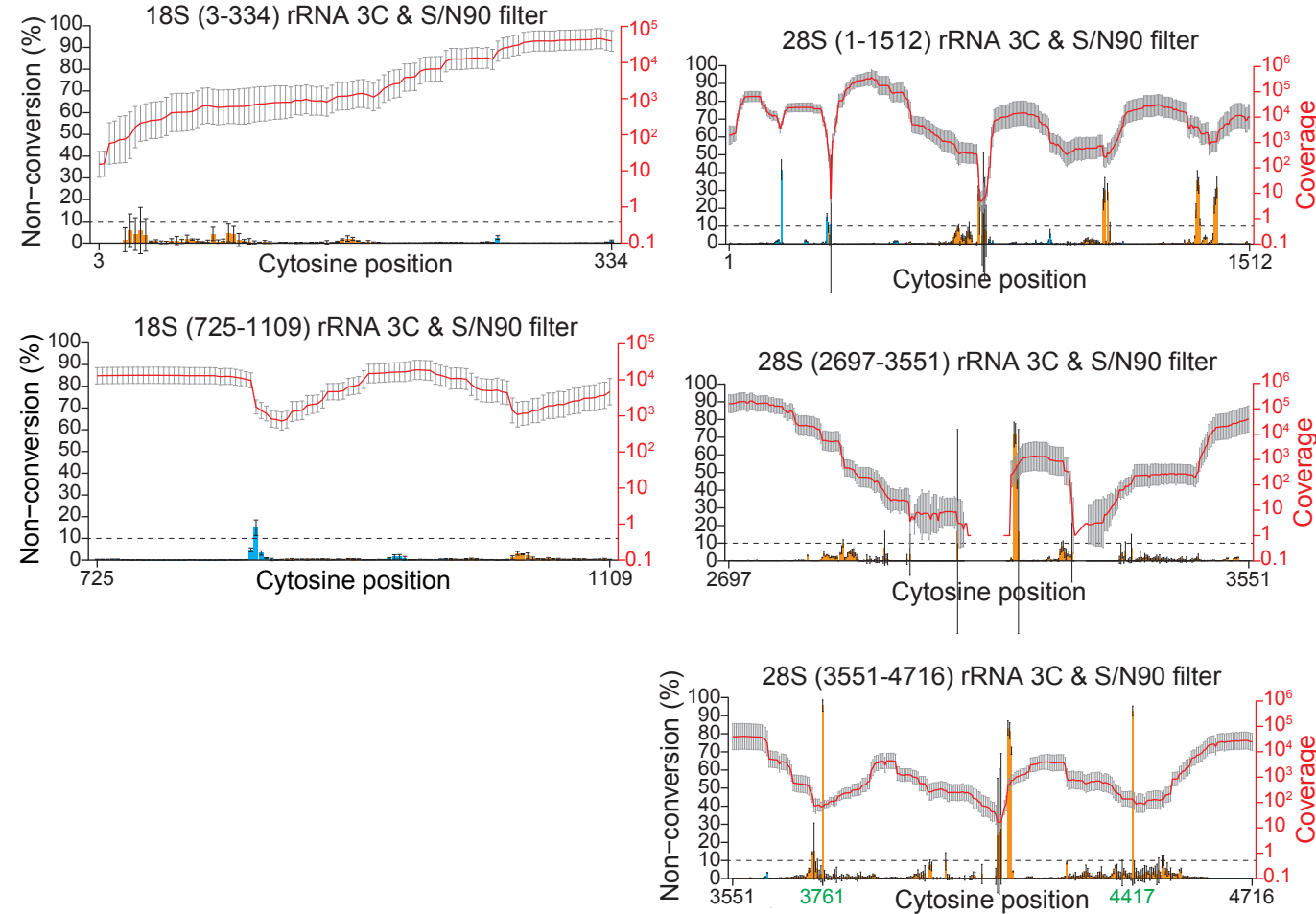

Supplement: Supplementary file 7 — Figure S3. Effects of the 3C and S/N90 filters on specificity and sensitivity of m5C candidate site detection. Related to Fig. 2. In each panel, plots are arranged vertically by RNA under investigation, and horizontally by the extent of sequential filtering (initial read mapping—after removing reads with > 3 non-converted cytosines ‘3C filter’—after suppressing sites below the chosen signal-to-noise threshold ‘3C & S/N90 filter’ [less than 90% of reads passing the 3C filter]). Dual y-axis plots show either cytosine conversion (A,C) or non-conversion (B) (left y-axis, blue bars) and read coverage (right y-axis, red line) against cytosine position in the respective reference sequence (x-axis). Data is shown as mean across the three biological replicates with error bars indicating ± standard deviation. Candidate sites disqualified by the S/N90 filter are identified by orange bars. The effects of the filters were evaluated using selected spike-in control (A), rRNA (B) and tRNA (C) sequences. A: Panel of spike-in controls, R-Luc RNA and two arbitrarily selected ERCC transcripts. B: Mature ribosomal RNA species. Note that cytosine non-conversion is plotted for improved visualisation. The fourth to sixth panels show zoomed-in plots of fully filtered 18S and 28S rRNA data. Residues of zoomed regions are indicated on the top and correspond to numbering in full-scale plots. Green arrows and position labelling indicate the two known m5C sites in 28S rRNA [96]. C: Selected tRNA examples. tRNAAsp (GUC), tRNAGlu (UUC) and tRNAGly (GCC) were chosen to represent different m5C positions within tRNAs and to illustrate the adverse effect of the chosen filters on tRNAs with > 3 modified cytosines. Cytosine numbering is according to the tRNA consensus structural positions. [file 12915_2020_769_MOESM7_ESM.pdf]

**Figure S5:**

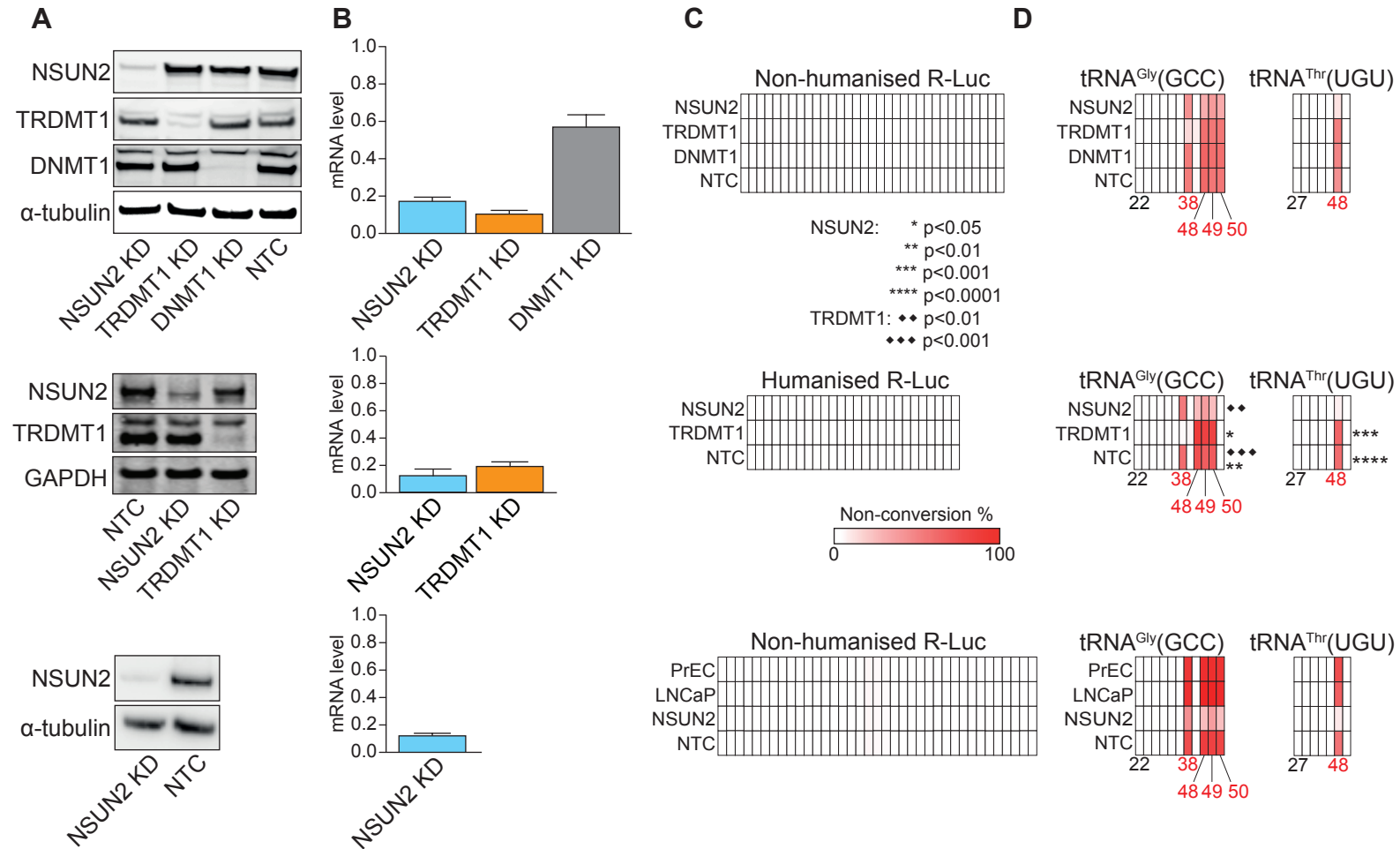

Supplement: Supplementary file 10 — Figure S5. siRNA knockdown efficiency controls. Related to Fig. 3. HeLa cells, or the prostate cell lines LNCaP or PrEC, were transiently transfected with siRNAs targeting the m5C:RNA methyltransferases NSUN2 or TRDMT1, the m5C:DNA methyltransferase DNMT1 or a non-targeting control (NTC) as indicated in the panels. Across panels, results are arranged with HeLa ‘confirmatory’ data on top (N = 1), HeLa ‘in depth’ data in the middle (N = 3), and prostate cell line ‘confirmatory’ data at the bottom (N = 1). A: Western blots for NSUN2, TRDMT1, DNMT1 and the internal controls alpha-tubulin or GAPDH are indicated on the left. siRNA knockdown condition is shown below. One replicate for the HeLa ‘in depth’ data is shown; similar results were obtained for the other replicates. B: mRNA levels are shown relative to those in the NTC control. HeLa RT-qPCR data were normalised to the internal control genes HPRT (top) or GAPDH (middle), RT-qPCR data from the prostate cell lines were normalised to the geometric mean of the internal control genes MRPL9, H2AFV and TCF25. ‘Confirmatory’ data (top and bottom) are shown as averages of three technical replicates. ‘In depth’ data (middle) are shown as averages of three biological replicates. Error bars indicate standard error of the mean. C-D: Amplicon-bsRNA-seq results for the R-Luc spike-in negative controls (C) and selected tRNAs as positive controls (D). Grids are organised by knockdown sample in rows and cytosine position along the analysed transcript section in columns (for tRNAs structural positions are given for the first interrogated cytosine as well as the candidate m5C sites [in red]). A white-to-red colour scale is used to tint each square by the degree of cytosine non-conversion. Note, C38 in tRNAGly (GCC) is a known target of TRDMT1, whereas C48–50 in tRNAGly (GCC) and C48 in tRNAThr (UGU) are mediated by NSUN2. Student’s t-test results of non-conversion change for tRNAGly (GCC) in the ‘confirmatory’ HeLa data is indicated ne [file 12915_2020_769_MOESM10_ESM.pdf]

Figure S6:

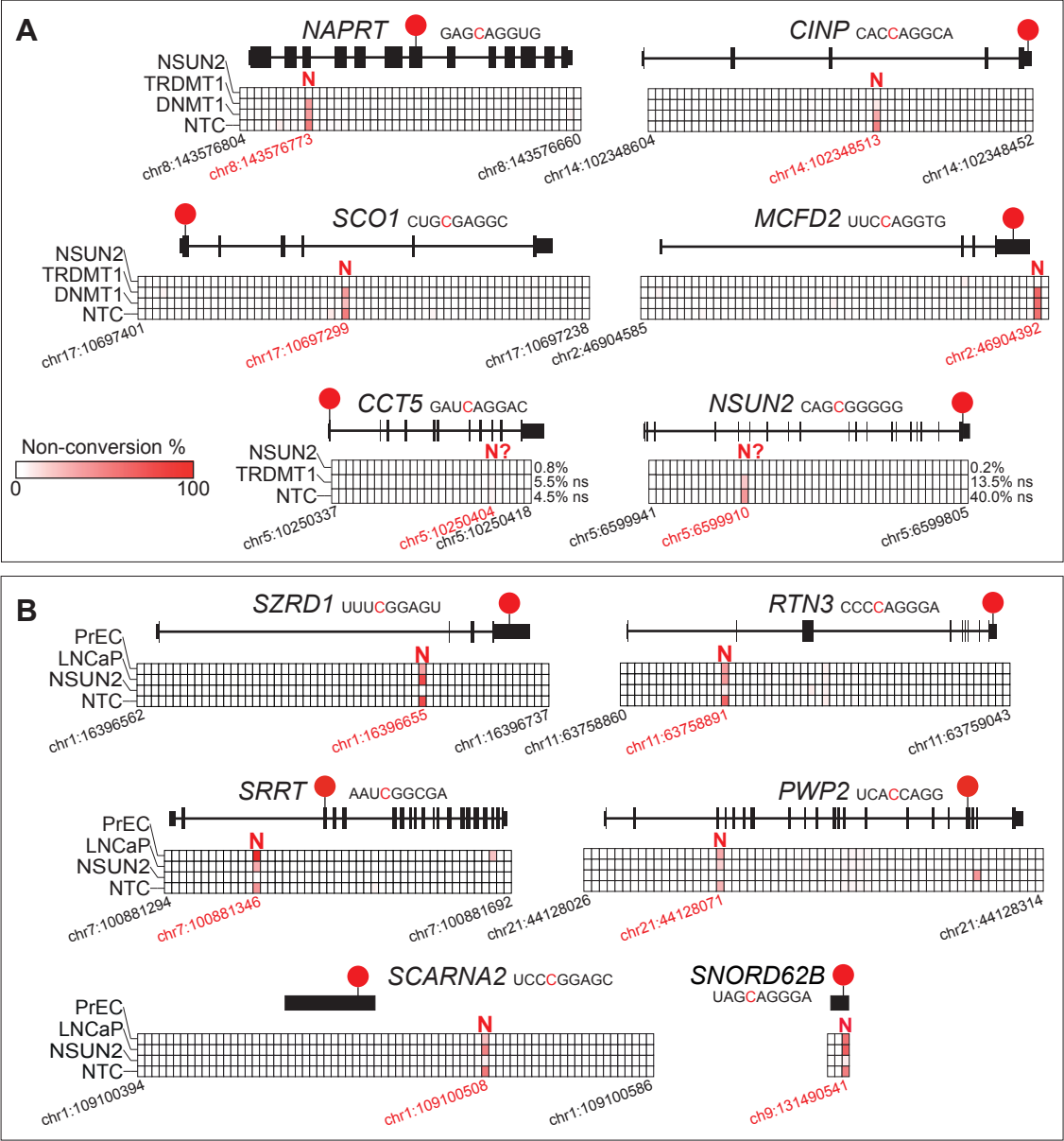

Supplement: Supplementary file 11 — Figure S6. Additional validation and NSUN2-dependence of candidate m5C sites in mRNA and ncRNA. Related to Fig. 3. Amplicon-bsRNA-seq was performed with total RNA isolated from HeLa cells, or the prostate cell lines LNCaP or PrEC, after siRNA-mediated m5C:RNA methyltransferase knockdown targeting NSUN2 or TRDMT1 along with control siRNAs (targeting m5C:DNA methyltransferase DNMT1 or a non-targeting control [NTC]; see Fig. S5 for knockdown efficiency controls). Read coverage per amplicon was from 3500 to 67,500 (see Table S6). Grids are organised by knockdown sample in rows and cytosine position along the analysed transcript section in columns (genomic coordinates are given for the first and last interrogated cytosine position [in black], as well as the candidate m5C site [in red]). A white-to-red colour scale is used to tint each square by the degree of cytosine non-conversion. The longest mRNA isoform (based on Ensembl) is shown with the candidate m5C site position indicated by a red circle. The sequence context (non-converted cytosine indicated in red) is given. The enzyme identified to be responsible for cytosine methylation is indicated above the candidate sites: N – NSUN2; N? – unresolved but likely NSUN2. A: ‘Confirmatory’ HeLa data (N = 1) shown for mRNA sites in NAPRT (nicotinate phosphoribosyltransferase), CINP (cyclin dependent kinase 2 interacting protein), SCO1 (SCO cytochrome C oxidase assembly protein 1) and MCFD2 (multiple coagulation factor deficiency 2) (top and middle row). ‘In depth’ data (N = 2–3) is shown as the average of at least two biological replicates for mRNA sites in CCT5 (chaperonin containing TCP1 Subunit 5) and NSUN2 (bottom row). These two sites are likely controlled by NSUN2, although the non-conversion change is not significantly (ns) different (Student’s t-test) in comparison to the NSUN2 KD sample (non-conversion averages are indicated to the right; see also Table S6). B: ‘Confirmatory’ prostate data (N = 1) shown for mRNA sites [file 12915_2020_769_MOESM11_ESM.pdf]

**Figure S7:**

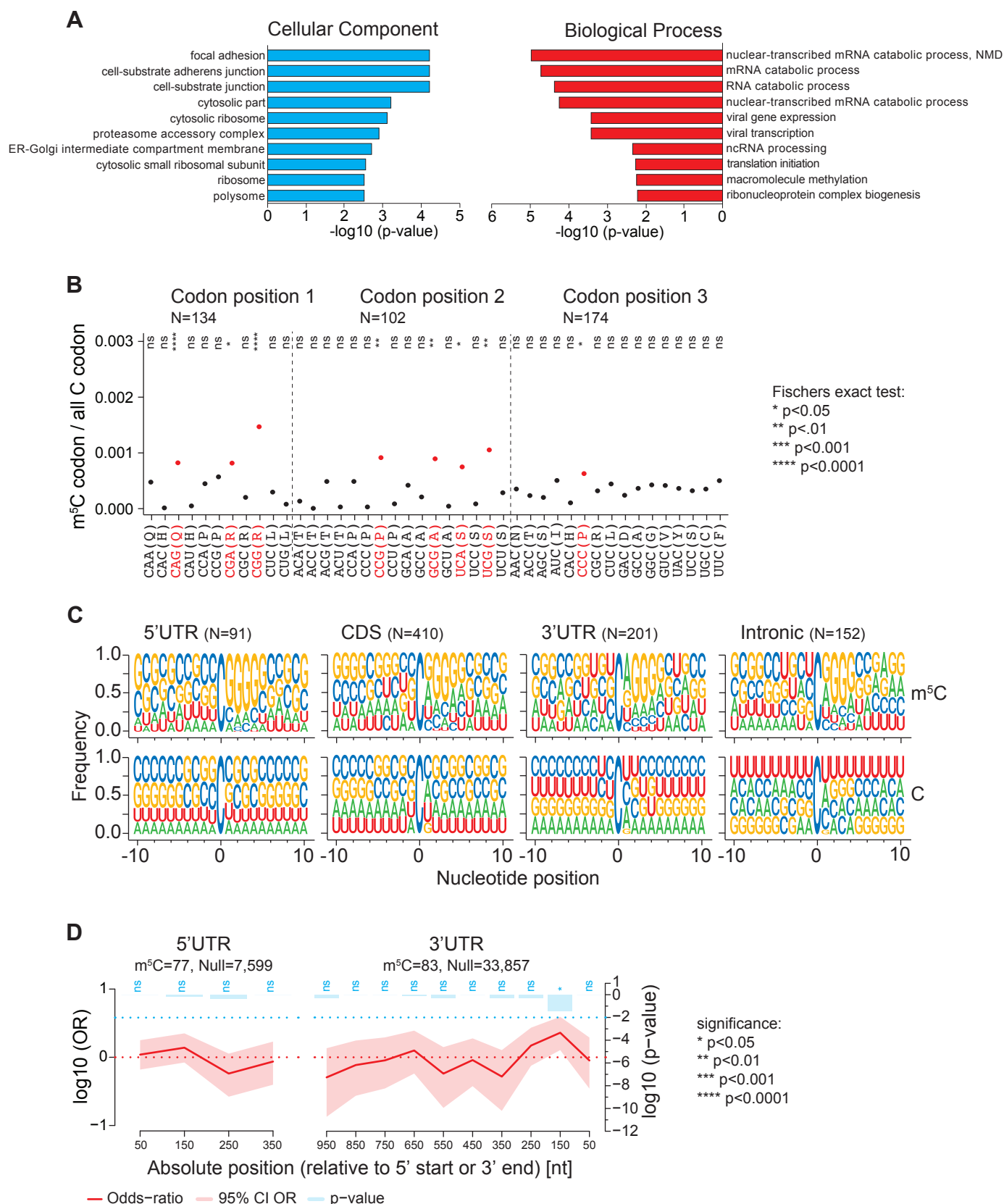

Supplement: Supplementary file 13 — Figure S7. Sequence context and NSUN2-dependence of candidate m5C sites. Related to Fig. 4. A: Gene Ontology (GO) term enrichment of candidate sites. Analysis was performed using the enrichGO function in ClusterProfiler using transcriptome-wide candidate sites (n = 846), with all genes detected at FKPM≥1 in the bsRNA-seq used as background. Bonferroni correction was applied and terms with p < 0.05 deemed enriched. The ten most enriched terms for Cellular Component (left) and Biological Process (right) are shown (for full list see Table S7). No enrichment was obtained for Molecular Function GO terms. B: Codon position enrichment analysis of candidate sites within the CDS of protein-coding genes. All three codon positions were analysed and are indicated at the top. Codons preferentially containing candidate sites are indicated in red, with significance following Fisher’s exact test indicated: ns - not significant. C: Sequence context of candidate sites (top) in comparison to all cytosines in the same transcripts (bottom). Logos were generated for candidate sites present within the four RNA transcript regions (5′UTR: m5C, N = 91; Null = 150,943. CDS: m5C, N = 410; Null = 1,139,649. 3′UTR: m5C, N = 201; Null = 589,922. Intronic: m5C, N = 152; Null = 26,326,026). All sites from ‘protein-coding’ (N = 846) and ‘NMD’ RNA biotypes (N = 8) were included. D: Spatial enrichment analyses of candidate sites within mRNAs. Site are placed into bins as indicated on the x-axis. Site distribution across bins is compared to matching randomised cytosine sampling (Null) and the log10 Odds-ratio (OR) is plotted as a red line with the 95% confidence interval (CI) shaded. Significance of enrichment is plotted as the log10 p-value by blue bars: ns - not significant. Analyses were anchored at either the transcription start (left) or the transcription end (right) and performed using RNAModR with a bin width of 100 nt and a window of 400 nt for the 5′UTR or 1000 nt for the 3′UTR region. [file 12915_2020_769_MOESM13_ESM.pdf]

**Figure S8:**

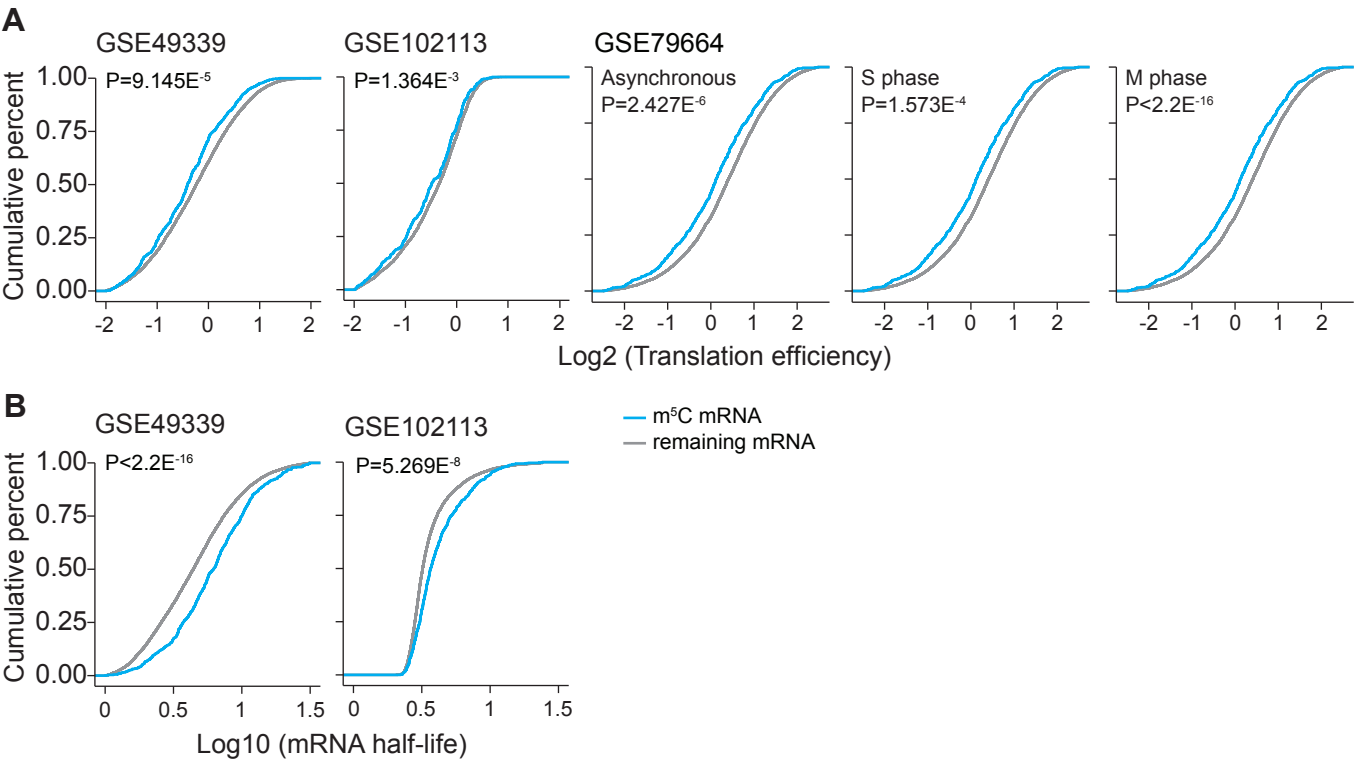

Supplement: Supplementary file 15 — Figure S8. Correlation of mRNA translation and stability with m5C site content. Related to Fig. 4. A: Cumulative density distribution of translation efficiency for candidate site-containing and all remaining mRNAs. Translation efficiency values are based on HeLa cell ribosome profiling data from [X Wang, ZK Lu, A Gomez, GC Hon, YN Yue, DL Han, Y Fu, M Parisien, Q Dai, GF Jia, et al. [33]; first plot], [[79]; second plot] or [[80]; cell cycle plots]. (GSE49339: m5C mRNA, N = 666; remaining mRNA, N = 12,185. GSE102113: m5C mRNA, N = 667; remaining mRNA, N = 11,460. GSE79664: m5C mRNA, N = 669; remaining mRNA, N = 11,717). B: Cumulative density distribution of mRNA half-life for candidate site-containing and all remaining mRNAs. HeLa cell mRNA half-life data was taken from [[33]; first plot)] or [[79]; second plot]. (GSE49339: m5C mRNA, N = 649; remaining mRNA, N = 10,569. GSE102113: m5C mRNA, N = 612; remaining, N = 11,175). [file 12915_2020_769_MOESM15_ESM.pdf]

Figure S9:

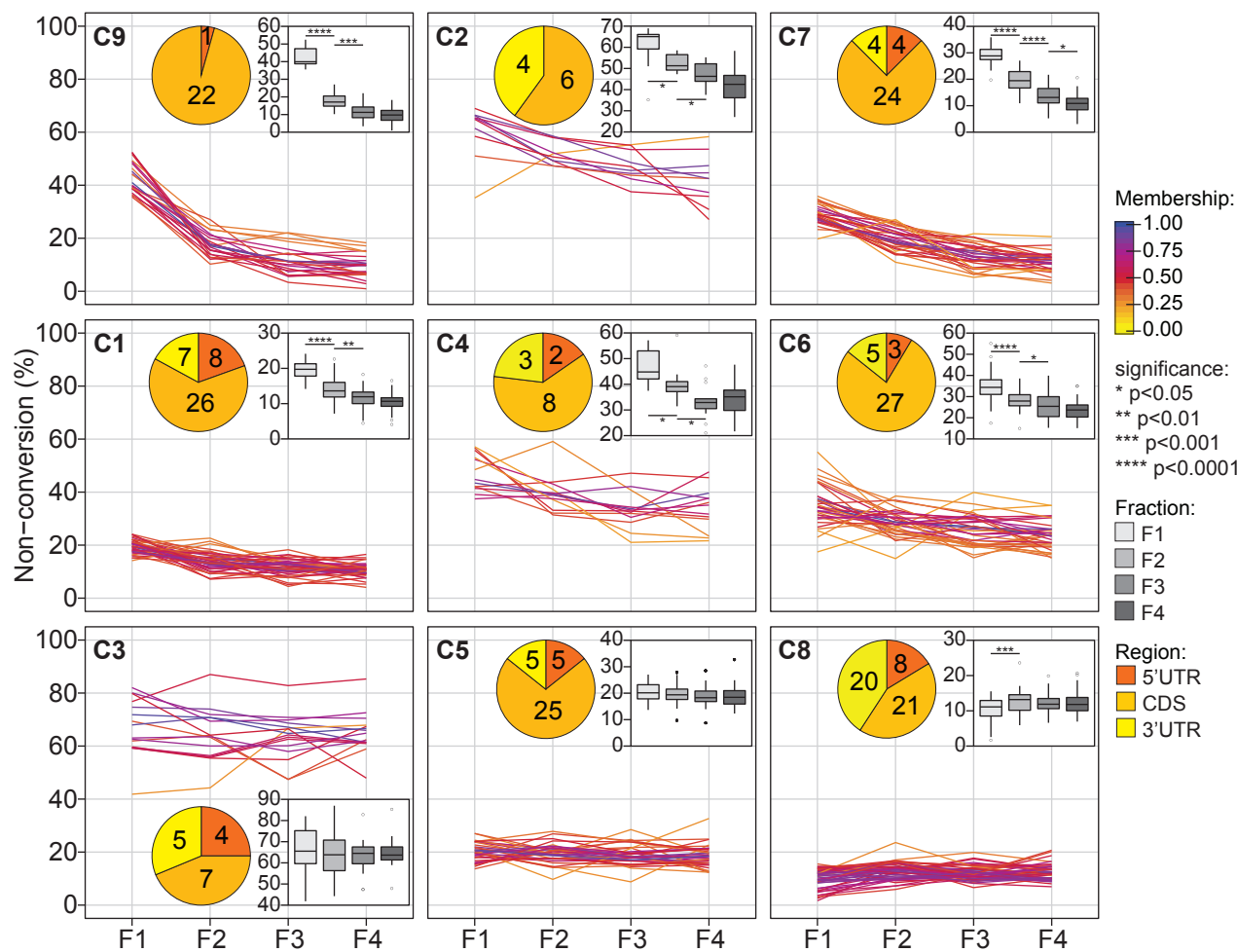

Supplement: Supplementary file 17 — Figure S9. Clustering of candidate m5C site non-conversion level patterns across the polysome profile. Related to Fig. 5. bsRNA-seq libraries were grouped as biological triplicates per fraction (e.g. LibB 1, LibC1 and LibE1 each report on sites detected in bsRNA-seq fraction 1), allowing the calculation of average non-conversion levels per individual site and per fraction. Non-conversion levels per individual site across the polysome profile were partitioned into nine soft clusters (C1–9 arranged here by similarity in trend with polysome profile) using Mfuzz. 254 candidate m5C sites were included based on having coverage in at least 9 out of 12 bsRNA-seq fraction samples and ≥ 10 average coverage in each of the four bsRNA-seq fractions. Degree of cluster ‘membership’ is indicated by the colour scale depicted to the right of panel A. Candidate sites with high membership (blue) have the best match to the respective cluster’s overall pattern. The legend to the right gives a colour/significance key applicable to all panels. Insets are: pie charts showing distribution of cluster members across different mRNA regions; boxplots showing site non-conversion distribution of cluster members across the polysome profile. Asterisks indicate significance p-value from unpaired, two-tailed Student’s t-test comparing the means of adjacent fractions. For Fig. 5, clusters C9,2,7,1,4,6 were combined into the negative trend category, clusters C3,5 into the neutral category, and cluster C8 formed the positive category. (PDF 944 kb) [file 12915_2020_769_MOESM17_ESM.pdf]

Figure S10:

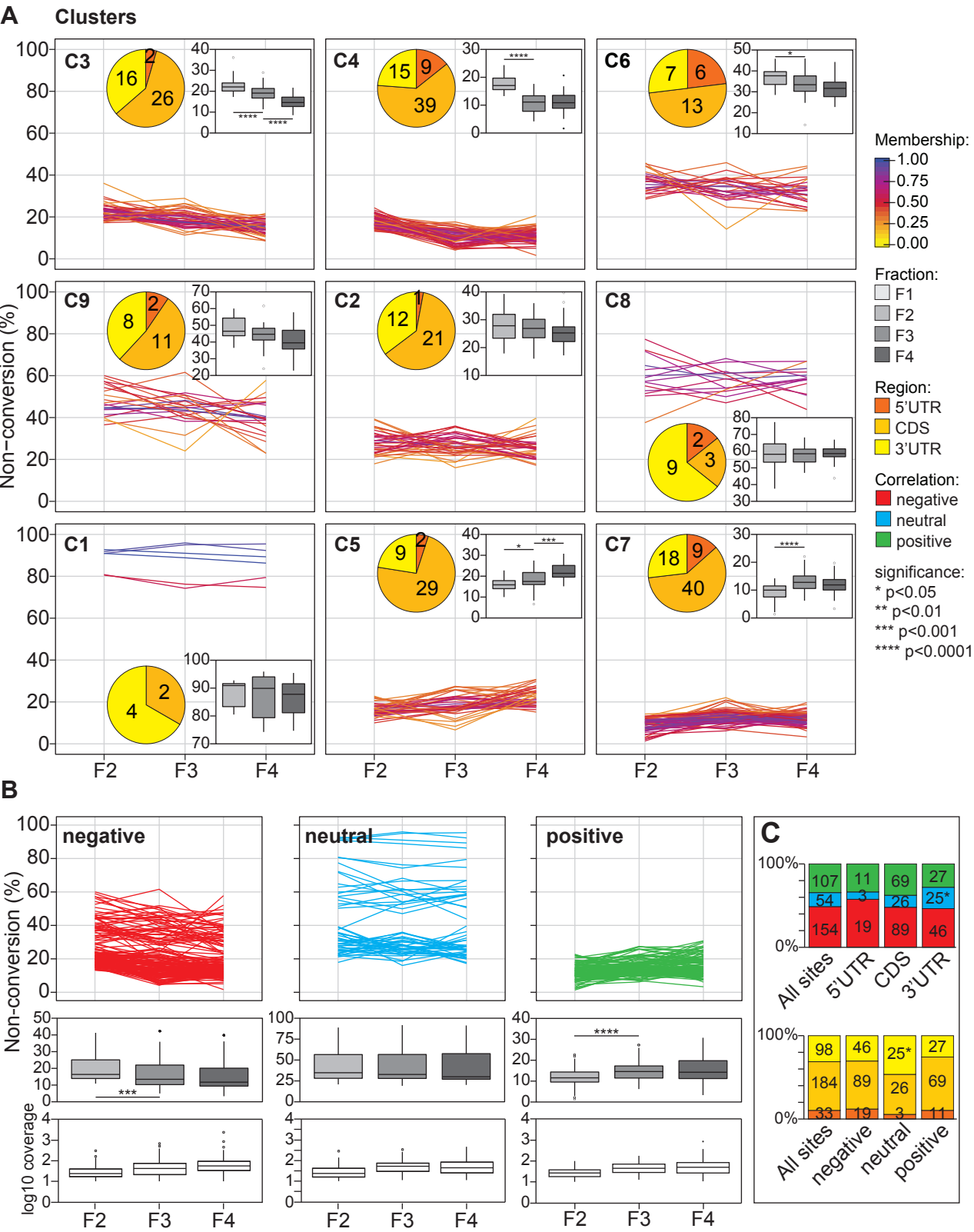

Supplement: Supplementary file 18 — Figure S10. Relationship between non-conversion level and mRNA translation state for candidate m5C sites with insufficient coverage in bsRNA-seq fraction 1. Related to Fig. 5. bsRNA-seq libraries were grouped as biological triplicates per fraction (e.g. LibB 1, LibC1 and LibE1 each report on sites detected in bsRNA-seq fraction 1), allowing the calculation of average non-conversion levels per individual site and per fraction. A: Non-conversion levels per individual site across the polysome profile were partitioned into nine soft clusters (C1–9 arranged here by similarity in trend with polysome profile) using Mfuzz. 315 candidate m5C sites were included based on having coverage in at least 9 out of 12 bsRNA-seq fraction samples and ≥ 10 average coverage in bsRNA-seq fractions 2–4 but failing this criterion for fraction 1. Degree of cluster ‘membership’ is indicated by the colour scale depicted to the right of panel A. Candidate sites with high membership (blue) have the best match to the respective cluster’s overall pattern. The legend to the right of panel A gives a colour/significance key applicable to all panels. Insets are: pie charts showing distribution of cluster members across different mRNA regions; boxplots showing site non-conversion distribution of cluster members across the polysome profile. Asterisks indicate significance p-value from unpaired, two-tailed Student’s t-test comparing the means of adjacent fractions. B: Mfuzz clusters from panel A were grouped into three translation state trend categories by visual inspection, showing a negative (clusters C3,4,6,9; N = 133), neutral (clusters C2,8,1; N = 75) or positive trend (clusters C5,7; N = 107) with polysome association. Top panels: line graphs displaying individual site average non-conversion levels across fractions. Middle panels: boxplots showing distribution of site non-conversion levels in each fraction. Asterisks indicate significance p-value from unpaired, two-tailed Student’s t-test comparing [file 12915_2020_769_MOESM18_ESM.pdf]

Figure S11:

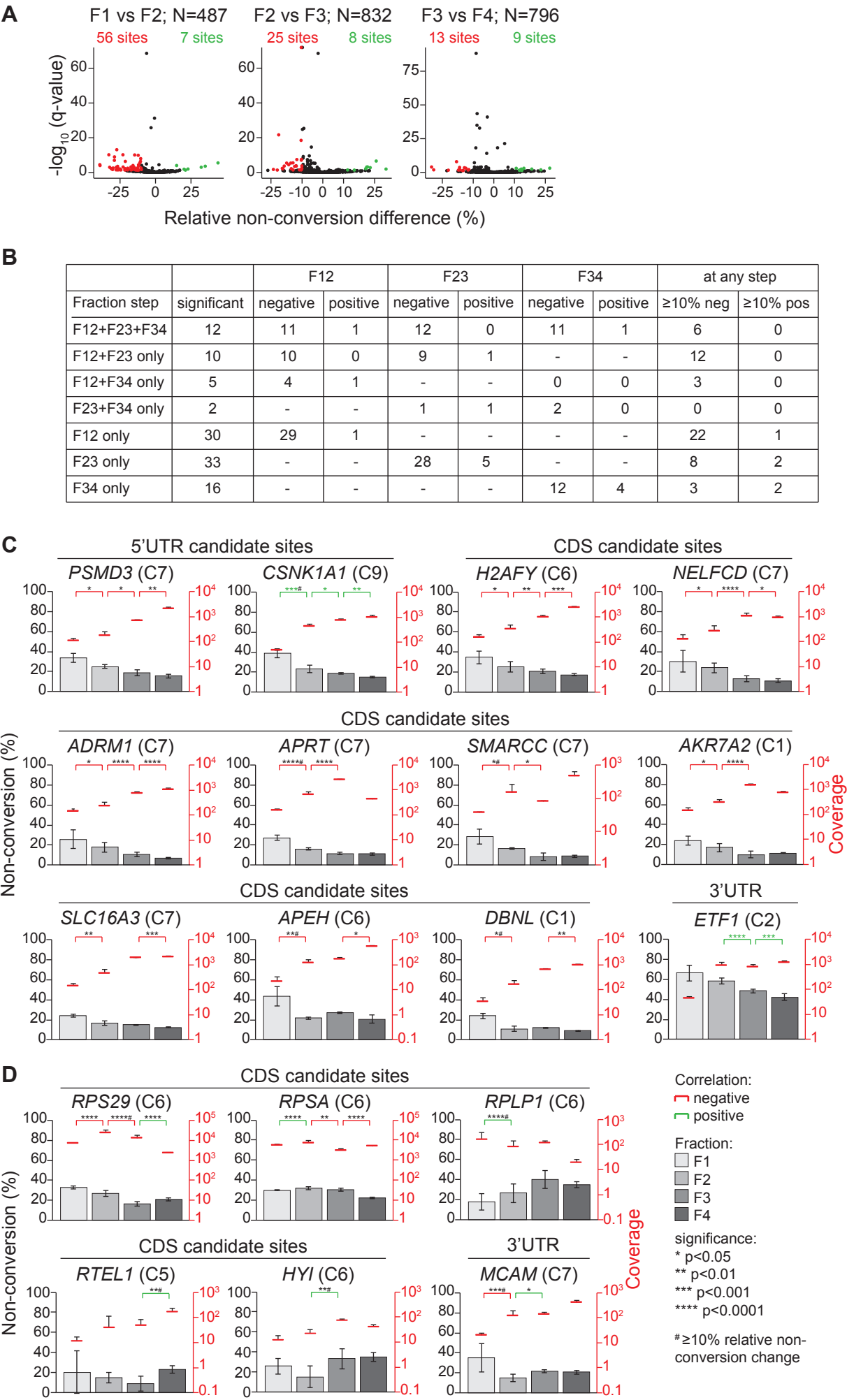

Supplement: Supplementary file 19 — Figure S11. Identification of individual sites with significant correlation of cytosine non-conversion with polysome co-sedimentation. Related to Fig. 6. A: Transcriptome-wide candidate m5C sites were used as input for logistic regression analysis (methylKit; [94]. For each pairwise comparison of adjacent polysome profile fractions a minimum of ≥10 average read coverage across each bsRNA-seq fraction was required. Plots show the -log10 q-value (FDR) of the non-conversion change, against the relative non-conversion difference for each pairwise comparison, with the number of sites that qualified indicated above (993 of 1034 sites were involved in at least one such comparison). Each dot represents a single site, sites that score as significant (q-value < 0.05) and showing a relative non-conversion change ≥10% are coloured in red (negative correlation) and green (positive correlation), respectively. B: Characteristics of sites included in the F1234 clustering (Figs. 5 and S9) that showed any significant trend as analysed in panel A. C-D: Examples of individual sites in mRNA showing significant correlation of cytosine non-conversion with translation state. Dual axis charts show cytosine non-conversion (bars) and coverage (red lines) for a given site across the polysome gradient. Data is shown as means across biological triplicates, with error bars indicating standard deviation. Asterisks indicate significance p-values after logistic regression testing (see key next to panel D). The gene name for each candidate site and its position within the mRNA is given. C: Individual examples with significant negative non-conversion change in at least two fraction steps. The cluster number from the F1234 clustering is indicated in brackets. Candidate sites shown here are from the following mRNAs: PSMD3 (proteasome 26S subunit); CSNK1A1 (casein kinase 1 alpha 1); H2AFY (H2A histone family member Y); NELFCD (negative elongation factor complex member C/D); ADRM1 (adhesion regulating mol [file 12915_2020_769_MOESM19_ESM.pdf]
