# Supplementary material for: Multiple links between 5-methylcytosine content of mRNA and translation
Source: BMC Biol. 2020 Apr 15;18:40. doi: 10.1186/s12915-020-00769-5 (PMC7158060; doi:10.1186/s12915-020-00769-5)
Supplement: Supplementary file 4 — Table S3. Primers used in this study. [file 12915_2020_769_MOESM4_ESM.docx]

**Supplementary Table S3: Primers used in this study.**

| **target transcript** |  | **primer sequence 5’-3’** | **experiment** |
| --- | --- | --- | --- |
| ***RPL13a*** | Forward | CCTGGAGGAGAAGAGGAAAGAGA | RT-qPCR |
|  | Reverse | TTGAGGACCTCTGTGTATTTGTCAA |  |
| ***RPS3*** | Forward | GCAAGATGGCAGTGCAAATA | RT-qPCR |
|  | Reverse | GGTTGGTGTAACTCGCACCT |  |
| ***MAP2K2*** | Forward | GTGCTTCTCTCGGAGGTACG | RT-qPCR |
|  | Reverse | TTTGCATGGAACACATGGAC |  |
| ***CCND1*** | Forward | ACGCGCAGACCTTCGTT | RT-qPCR |
|  | Reverse | AGCGTGTGAGGCGGTAGTA |  |
| ***SZRD1*** | Forward | TCTTAGGGGTCTGCAGTGCT | RT-qPCR |
|  | Reverse | AGCCTAGGCCTCTGAGGAAC |  |
| ***NDUFB7*** | Forward | ccttcatGcGcatcacataG | RT-qPCR |
|  | Reverse | GtGacaGcttccccaacttc |  |
| ***R-Luc*** | Forward | GGCGAGAAAATGGTGCTTGAG | RT-qPCR |
|  | Reverse | TCCTTGAATGGCTCCAGGTAGG |  |
| ***NSUN2*** | Forward | GGGCTCAGCTGTGTGCTT | RT-qPCR |
|  | Reverse | TGGAATAAACGTCAGCCAAA |  |
| ***TRDMT1*** | Forward | CGCTGTTTCACTGTTATCTTCTCA | RT-qPCR |
|  | Reverse | TCCTAAAGAAATAGCAAATCTCCTTG |  |
| ***DNMT1*** | Forward | GGTTTGCCTGGTGCTTTTC | RT-qPCR |
|  | Reverse | CCCAAGTAACTGGGATTAGAGC |  |
| ***GAPDH*** | Forward | CAAGAGCACAAGAGGAAGAGA | RT-qPCR |
|  | Reverse | AACTGTGAGGAGGGGAGATT |  |
| ***HPRT*** | Forward | TGACACTGGCAAAACAATGCA | RT-qPCR |
|  | Reverse | GGTCCTTTTCACCAGCAAGCT |  |
| ***MRPL9*** | Forward | cacctcacaccaatactcgc | RT-qPCR |
|  | Reverse | aaatagttgcccgccacttc |  |
| ***H2AFY*** | Forward | ttgaagactcgcaccacaag | RT-qPCR |
|  | Reverse | gaagcattacctgccagctc |  |
| ***TCF25*** | Forward | caacgtgtgtaccccaagtg | RT-qPCR |
|  | Reverse | caaacgcaaagaaggagagg |  |
| ***tRNA^Gly^(GCC)*** | Forward | gTATggGTGgTTTAgTGGTA | amplicon bsRNA-seq |
|  | Reverse | CATAAACCAAAAATCAAACCC |  |
| ***tRNA^Thr^(UGU)*** | Forward | GTTTTATAGTTTAGGGGTTA | amplicon bsRNA-seq |
|  | Reverse | CCCCAACAAAAATTAAACTC |  |
| ***Humanised R-Luc*** | Forward | GTTTATGTTGAGAGTGTTGTGG | amplicon bsRNA-seq |
|  | Reverse | CTCCAATTTCCACATAATCTTAC |  |
| ***R-Luc*-1** | Forward | TGATGAAATGGGAAAATATAT | amplicon bsRNA-seq |
|  | Reverse | CTTTAAAAAACCTCCCACACCT |  |
| ***R-Luc*-2** | Forward | GAAGAAGGAGAAAAAATGGTT | amplicon bsRNA-seq |
|  | Reverse | CATTATAATTCCTAACAATTT |  |
| ***RPS3*** | Forward | GAAAtTttGAGGAtAGAGGG | amplicon bsRNA-seq |
|  | Reverse | CCACAATaCTCACaTaaTC |  |
| ***NDUFB7*** | Forward | AAGGAGTGTGAGATGGTGG | amplicon bsRNA-seq |
|  | Reverse | CTCCCaCTCAAACTCCTTCAT |  |
| ***RTN3*** | Forward | GGGAGTTAGTAGGTATGTTG | amplicon bsRNA-seq |
|  | Reverse | CAACAATACACAACAATTACAA |  |
| ***SZRD1*** | Forward | TGTAAGAAAAAGTGTATGGG | amplicon bsRNA-seq |
|  | Reverse | CTCATCTATCCTAACAACTA |  |
| ***OSBPL8*** | Forward | GTGGATTTTGGGGTTGAAAG | amplicon bsRNA-seq |
|  | Reverse | CCCATACAAAACTCCCAAAA |  |
| ***NAPRT*** | Forward | TGGTGAGGGtttTGGGGTGGA | amplicon bsRNA-seq |
|  | Reverse | ACCACTCCTCCACACaCTaTA |  |
| ***SCO1*** | Forward | GAGTTTTGGGGtttAGttG | amplicon bsRNA-seq |
|  | Reverse | CTAAAaACTTCCAaaAAACAaaC |  |
| ***CINP*** | Forward | TATGTGGAGAGtGAtAGtAGG | amplicon bsRNA-seq |
|  | Reverse | CTCTaaCCAaAAaACCCCAA |  |
| ***MCFD2*** | Forward | GGGGTTAtAGGGtttATGtAAG | amplicon bsRNA-seq |
|  | Reverse | CCACCAACAaCTTaCACCaT |  |
| ***CAGE1*** | Forward | GAAAtTtTGAGGAtAGAGAG | amplicon bsRNA-seq |
|  | Reverse | CCACaATaCTCACaTaaTC |  |
| ***NSUN5P2*** | Forward | GtAAAAAGtTGtAGttGGTG | amplicon bsRNA-seq |
|  | Reverse | CTaCTaCCTaCaaTaAaaaCC |  |
| ***RPPH1*** | Forward | TGAGTTTTGGGGAGGTGAGTT | amplicon bsRNA-seq |
|  | Reverse | CCATTAAACTCACTTCACT |  |
| ***SNORD62B*** | Forward | GATTTTTTTGATTTTTTATTGTGG | amplicon bsRNA-seq |
|  | Reverse | CTCTCAATCACTATCCTCTT |  |
| ***SCARNA2*** | Forward | TTTTATTTGATTGGATTGTG | amplicon bsRNA-seq |
|  | Reverse | CAAAATCACCTCAATAATCA |  |
| ***GIPC1*** | Forward | GTGTATTAAGGAGGGTAGTGTG | amplicon bsRNA-seq |
|  | Reverse | AACCTTACAAAACTCCATAAACTTC |  |
| ***GID8*** | Forward | GAAAAATGGTTTTTGTAGGTAGTGG | amplicon bsRNA-seq |
|  | Reverse | CAACAACCAAAAACCCCCAAAC |  |
| ***PIGG*** | Forward | GTGTGGTAGGATGGGTTTGG | amplicon bsRNA-seq |
|  | Reverse | CCAACAACAAACCAATATTAACCAC |  |
| ***ZDHHC8*** | Forward | TGGGTTTTGTGTTGTGTTTAG | amplicon bsRNA-seq |
|  | Reverse | CCACCACATCCCTCCCC |  |
| ***CCT5*** | Forward | GGGGGAAGTAATTTTGGTTGTTG | amplicon bsRNA-seq |
|  | Reverse | AAAAACCTCAAATCCCATAAAACA |  |
| ***NSUN2*** | Forward | GGTTGTGATTTGGTGGGGG | amplicon bsRNA-seq |
|  | Reverse | CTCCTATAATCCCACCAATCTAC |  |
| ***PWP2*** | Forward | GTTTGAGTTGGATATTAGTG | amplicon bsRNA-seq |
|  | Reverse | CCAAATAACAAAACACTTC |  |
| ***SRRT-1*** | Forward | GGATGATTGGAATGATAGAG | amplicon bsRNA-seq |
|  | Reverse | CCAACATAAAACATCTCATAAC |  |
| ***SRRT-2*** | Forward | GATTGGAATGATAGAGAGTGGG | amplicon bsRNA-seq |
|  | Reverse | TCATCCCAATCTCTCCTCATAC |  |
