# Supplementary material for: Multiple links between 5-methylcytosine content of mRNA and translation
Source: BMC Biol. 2020 Apr 15;18:40. doi: 10.1186/s12915-020-00769-5 (PMC7158060; doi:10.1186/s12915-020-00769-5)
Supplement: Supplementary file 9 — Figure S4. m5C candidate site call reproducibility across biological replicates and effects of non-conversion ‘noise suppression’. Related to Fig. 2. A: Pair-wise scatter plot comparisons of transcriptome-wide candidate sites called in composite libraries of each biological replicate. Sites shown passed the 80CT, 30RC and 5C filter in their respective composite library (a non-conversion cut-off was not applied). Further to that, only sites with coverage in all three replicates were used. The adjusted R-squared value following linear regression is shown. B: Effect of the 3C and S/N90 filters on candidate site calling in different RNA types. The number of candidate sites that passed the 80CT, 30RC, 5C filter in their respective composite library and fulfilled the 10MM criterion are listed. C: Position of candidate sites in the tRNA cloverleaf consensus structure. Each circle indicates a nucleotide position within the tRNA cloverleaf structure, with blue filled circles indicating position at which candidate sites were identified. Iso-acceptors found to carry the candidate site are identified by the single letter amino acid code. D: Genetic code table highlighting tRNA iso-decoders with candidate sites in blue. C-D: Of note, we detected the NSUN2-dependent sites at the edge of the variable loop at position C48–50 in a variety of tRNA iso-decoders, as well as at position C34 of intron-containing tRNALeu (CAA). We further identified the TRDMT1-dependent modification of C38 in tRNAAsp (GUC). Interestingly, we also detected several candidate sites at structural position C72. The established NSUN6-dependent sites in tRNAThr (UGU) and tRNACys (GCA) iso-decoders did not receive read coverage. Instead, we saw clear non-conversion at C72 in tRNAIle (UAU), tRNALys (CUU) and tRNASer (ACU); these might be novel NSUN6 substrates. C70 in tRNAGly (CCC) is indicated in the figure, however, detection of this site is heavily driven by the terminal base of reads in one direction, thus lik [file 12915_2020_769_MOESM9_ESM.pdf]

Figure S4:

A 3C & S/N90 and 80CT, 30RC, 5C

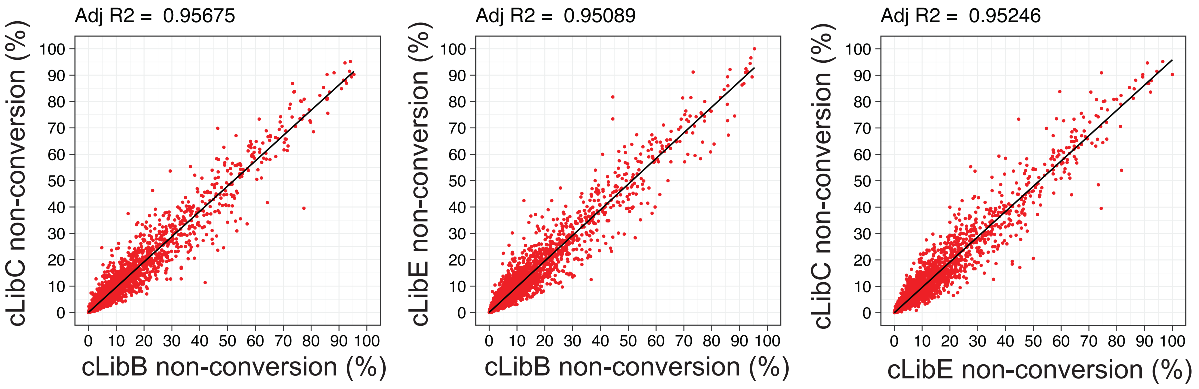

B 80CT, 30RC, 5C, 10MM

candidate sites	Raw data	3C filter	3C & S/N90 filter
rRNA	885	34	5
tRNA	177	156	119
spike-ins	0	0	0
transcriptome	8,250	1,808	1,034

C

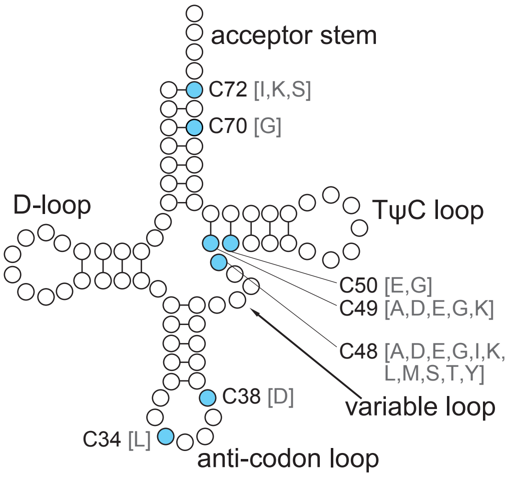

D

	A		G		U		C		
A	AAA	tRNA^{Phe}	AGA		AUA	tRNA^{Tyr}	ACA	tRNA^{Cys}	
G	GAA		GGA	tRNA^{Ser}	GUA		GCA		A
U	UAA	tRNA^{Leu}	UGA		UUA	tRNA^{Sup}	UCA	tRNA^{Sup}	
C	CAA		CGA		CUA		CCA	tRNA^{Trp}	
A	AAG		AGG		AUG	tRNA^{His}	ACG		
G	GAG	tRNA^{Leu}	GGG	tRNA^{Pro}	GUG		GCG	tRNA^{Arg}	G
U	UAG		UGG		UUG	tRNA^{Gln}	UCG		
C	CAG		CGG		CUG		CCG		
A	AAU		AGU		AUU	tRNA^{Asn}	ACU	tRNA^{Ser}	
G	GAU	tRNA^{Ile}	GGU	tRNA^{Thr}	GUU		GCU		U
U	UAU		UGU		UUU	tRNA^{Lys}	UCU	tRNA^{Arg}	
C	CAU	tRNA^{Met}	CGU		CUU		CCU		
A	AAC		AGC		AUC	tRNA^{Asp}	ACC		
G	GAC	tRNA^{Val}	GGC	tRNA^{Ala}	GUC		GCC	tRNA^{Gly}	C
U	UAC		UGC		UUC	tRNA^{Glu}	UCC		
C	CAC		CGC		CUC		CCC		
